# IL-6-Mediated Activation of Stat3α Prevents Trauma/Hemorrhagic Shock-Induced Liver Inflammation

**DOI:** 10.1371/journal.pone.0021449

**Published:** 2011-06-29

**Authors:** Ana Moran, Stephen A. Thacker, Ayse Akcan Arikan, Mary-Ann A. Mastrangelo, Yong Wu, Bi Yu, David J. Tweardy

**Affiliations:** 1 Infectious Diseases Section, Department of Medicine, Baylor College of Medicine, Houston, Texas, United States of America; 2 Critical Care Section, Department of Pediatrics, Baylor College of Medicine, Houston, Texas, United States of America; 3 Department of Molecular, Cellular Biology, Baylor College of Medicine, Houston, Texas, United States of America; 4 Pediatric Infectious Diseases Section, Department of Pediatrics, Baylor College of Medicine, Houston, Texas, United States of America; Ohio State University, United States of America

## Abstract

Trauma complicated by hemorrhagic shock (T/HS) is the leading cause of morbidity and mortality in the United States for individuals under the age of 44 years. Initial survivors are susceptible to developing multiple organ failure (MOF), which is thought to be caused, at least in part, by excessive or maladaptive activation of inflammatory pathways. We previously demonstrated in rodents that T/HS results in liver injury that can be prevented by IL-6 administration at the start of resuscitation; however, the contribution of the severity of HS to the extent of liver injury, whether or not resuscitation is required, and the mechanism(s) for the IL-6 protective effect have not been reported. In the experiments described here, we demonstrated that the extent of liver inflammation induced by T/HS depends on the duration of hypotension and requires resuscitation. We established that IL-6 administration at the start of resuscitation is capable of completely reversing liver inflammation and is associated with increased Stat3 activation. Global assessment of the livers showed that the main effect of IL-6 was to normalize the T/HS-induced inflammation transcriptome. Pharmacological inhibition of Stat3 activity within the liver blocked the ability of IL-6 to prevent liver inflammation and to normalize the T/HS-induced liver inflammation transcriptome. Genetic deletion of a Stat3β, a naturally occurring, dominant-negative isoform of the Stat3, attenuated T/HS-induced liver inflammation, confirming a role for Stat3, especially Stat3α, in preventing T/HS-mediated liver inflammation. Thus, T/HS-induced liver inflammation depends on the duration of hypotension and requires resuscitation; IL-6 administration at the start of resuscitation reverses T/HS-induced liver inflammation, through activation of Stat3α, which normalized the T/HS-induced liver inflammation transcriptome.

## Introduction

Trauma complicated by hemorrhagic shock (T/HS) is the leading cause of death for those under 45 years old in the United States [1]. Initial survivors of T/HS are particularly susceptible to developing a systemic inflammatory response that triggers multiple organ failure (MOF), a therapeutic challenge and the leading cause of death among these patients [2], [3]. MOF is thought to be caused, at least in part, by excessive or maladaptive activation of inflammatory pathways [3]-[5]. The liver is one of the organs most frequently affected by T/HS, and its central role in metabolism and homeostasis makes this organ a critical one for survival of the host after severe injury [6], [7].

We have previously demonstrated that T/HS in a rodent model results in liver injury as evidenced by liver necrosis and inflammation [8], apoptosis [9] and elevated transaminases [10], and that administration of IL-6 at the start of resuscitation prevented liver necrosis and apoptosis [8], [9]. However, the contribution of the severity of HS to the extent of liver injury, whether or not resuscitation is required and the mechanism(s) for the IL-6 protective effect have not been explored. In the studies reported herein, we demonstrated that the extent of liver inflammation induced by T/HS depends on the duration of hypotension and requires resuscitation. We established that IL-6 administration at the start of resuscitation completely prevents liver inflammation and is associated with increased Stat3 activation. Microarray analysis of the livers showed that the main effect of IL-6 was to normalize the T/HS-induced inflammation transcriptome. Pharmacological inhibition of Stat3 activity within the liver blocked the ability of IL-6 to prevent liver inflammation and to normalize the T/HS-induced liver inflammation transcriptome. Genetic deletion of a Stat3β, a naturally occurring, dominant-negative isoform of the Stat3, attenuated T/HS-induced liver inflammation, confirming a role for Stat3, especially Stat3α, in preventing T/HS-mediated liver inflammation.

## Methods

### 

#### Ethics Statement

Animal studies were approved by the Baylor College of Medicine Institutional Review Board for animal experimentation and conform to National Institutes of Health guidelines for the care and use of laboratory animals (Protocol Approval ID: AN-1980). All animals were sacrificed under general anesthesia as part of our shock protocol to ameliorate suffering.

#### Rat and mouse protocols for trauma plus hemorrhagic shock

Adult male Sprague-Dawley rats were obtained from Harlan (Indianapolis, IN). Stat3β homozygous-deficient (Stat3β ^Δ/Δ^) mice were generated as described [11] and re-derived at Jackson labs. Pups from heterozygous matings were tailed and genotyped by PCR, as described, with minor modifications [11].

For the rat experiments in this study, 8-week old male Sprague-Dawley rats (200–250 gm) were used. Rats were subjected to the sham or T/HS protocols, as described [9], [12], [13] with modifications. Blood was withdrawn into a heparinized syringe episodically to maintain the target MAP at 35 mmHg until blood pressure compensation failed. Blood was then returned as needed to maintain the target MAP. The amount of shed blood returned (SBR) defined 5 different levels of shock severity reflected in the duration of hypotension: 0% SBR (SBR0) represented the lowest level of shock severity (duration of hypotension, 78±2.5 minutes), 10% SBR (SBR10; duration of hypotension, 149±41.4 minutes), 20% SBR (SBR20; duration of hypotension, 165±32.7 minutes), 35% SBR (SBR35; duration of hypotension, 211±7.6 minutes), and 50% SBR (SBR50; duration of hypotension, 273±24.9 minutes). At the end of the hypotensive period, rats were resuscitated as described [9], [12], [13] and humanely sacrificed 60 minutes after the start of resuscitation in order to capture the first wave of transcriptional changes. Where indicated, rats received 10 µg/kg of recombinant human IL-6 in 0.1 ml PBS at the initiation of the resuscitation or PBS alone. Sham rats were anesthetized and cannulated for 250 minutes but were not subjected to hemorrhage or resuscitation. One group of rats (UHS) was subjected to the most severe hemorrhagic shock protocol (50% SBR), but not resuscitated and kept at the target MAP (35 mmHg) for an additional 60 minutes (duration of hypotension  = 336±10.3 minutes) before sacrifice.

In the mice experiments, Stat3β^Δ/Δ^ mice and wild-type littermate mice were subjected to a T/HS protocol [9], [14], which was similar to the rat protocol except that the target MAP in the mouse was 30 mm Hg and the duration of hypotension was 300 min in all mice. Sham mice were anesthetized and immobilized in a pair-wise fashion with T/HS mice and sacrificed at the same time as their T/HS companion.

Rat and mouse livers were harvested immediately after sacrifice. The right liver lobe was fixed with paraformaldehyde solution (2%) for histological analysis and the left lobe was snap frozen in liquid nitrogen for protein and RNA extraction.

#### In vivo pharmacological inhibition of Stat3

To achieve pharmacological inhibition of Stat3 activity within the livers, rats were randomized to receive by tail vein injection the G-rich, quartet-forming oligodeoxynucleotide (GQ-ODN) T40214 or non-specific (NS)-ODN (2.5 mg ODN/kg) complexed in polyethyleneimine, as described [15], 24 hours prior to subjecting them to the SBR50 protocol with IL-6 treatment. The half-life of T40214 in tissues is ≥48 hr [16].

#### Myeloperoxidase (MPO) staining

To detect neutrophil (PMN) infiltration, paraffin-embedded liver sections were rehydrated from Xylene to PBS through a series of decreasing concentrations of ethanol, steamed in citrate buffer and the placed on a DAKO autostainer. Polyclonal rabbit MPO antibody-1 (Lab Vision Corporation) was used as instructed by the manufacturer. Slides were counterstained with hematoxylin as described before [14]. MPO positive cells were assessed microscopically by counting MPO positive cells in 20 random ×1000 high power fields by an experienced histologist blinded to the treatment each rat received. Data is presented as number of MPO positive cells per high power field.

#### Immunoblotting

Levels of Stat3 activation within the livers of rats were assessed by immunoblotting using whole-tissue extracts of liver sections with mouse monoclonal antibodies to Tyr705 phosphorylated (p)Stat3 (Cell Signaling Technology, Inc., Danvers, MA; 1∶1000 dilution for each antibody). Briefly, frozen livers were cut by cryotome into 5-micron sections and resuspended in cell lysis buffer for pStat3 detection (Cell Death Detection ELISA^plus^ Kit, Roche Diagnostics, Manheim, Germany). The supernatant was sonicated in ice 3 times, 10 seconds each. Samples were then centrifuged and the supernatant evaluated by Bradford assay for total protein quantification. Protein samples (60ug total protein) were separated by SDS-PAGE and transferred to a PVDF membrane. To detect pStat3, the membrane was incubated overnight with specific mouse monoclonal antibody and subsequently incubated with goat anti-mouse antibody with horseradish peroxidase (HRP) conjugate (Zymed, San Francisco, CA) for 1 hour. ECL agent (Amersham Biosciences, UK) was used for detection. The membrane was then stripped (using RestoreTM Western Blot Stripping Buffer, PIERCE, Rockford, IL) and immuoblotting performed to detect total Stat3 protein using mouse IgG monoclonal antibody to Stat3 (BD Biosciences, Rockville, MD; 1∶1000 dilution) as described before [9], [12], [13]. Detection was done using ECL agent. Densitometry was performed using ImageQuant TL v2005 software (Amersham Biosciences, Buckinghamshire, England). Results are expressed as the ratio of pStat3 (after background signal subtraction) to total Stat3 signal (after background signal subtraction) for each sample.

#### RNA isolation and microarray hybridization and analysis procedures

Total RNA was isolated from 4–5 micron cryotome sections of liver using TRIzol® Reagent (Invitrogen, Carlsbad, California) single step RNA isolation protocol followed by purification with RNeasy® Mini Kit (QIAGEN, Hilden, Germany) as instructed by the manufacturer. Gene expression profiling was performed with the Affymetrix Rat Array RAE 230A following Affymetrix protocols used within the Baylor College of Medicine Microarray Core Facility.

#### Microarray Analysis

We used Affymetrics GCOS, dChip and Array Analyzer (Insightful Corporation) software packages for quality assessment and statistical analysis and annotation. Expression estimation and group comparisons were done with Array Analyzer. Low-level analyses included background correction, quartile normalization and expression estimation using GCRMA [17]. One-way analysis of variance (ANOVA) with contrasts [18] was used for group comparisons on all genes and on the list of inflammation-related genes only. P-values were adjusted for multiple comparisons using the Benjamini-Hockberg method [19] with S+Array Analyzer software as described [9], [12], [13]. The adjusted p-values represent false discovery rates (FDR) and are estimates of the proportion of “significant” genes that are false or spurious “discoveries”. We used a FDR = 10% as cut-off. All microarray data are MIAME-compliant, and relevant array data has been uploaded to Gene Expression Omnibus (GEO) with the following series accession number: GSE27978.

#### Statistical Analysis

Data are presented as mean ± standard error of the mean (SEM) unless otherwise indicated where appropriate. Multiple group comparisons of means were done by one-way analysis of variance (ANOVA). Post hoc analysis was done by Student-Newman-Keuls test for 2-group comparisons of means. Correlation between duration of hypotension and MPO positive cells/hpf was done for each individual study animal by Pearson correlation coefficient. Goodness of fit was evaluated by R-square. All statistical analyses were done on SigmaStat 2.03 (SPSS Inc., Chicago, IL).

## Results

### 

#### T/HS-induced liver inflammation depends on the severity of shock and requires resuscitation

**Figure 1:**
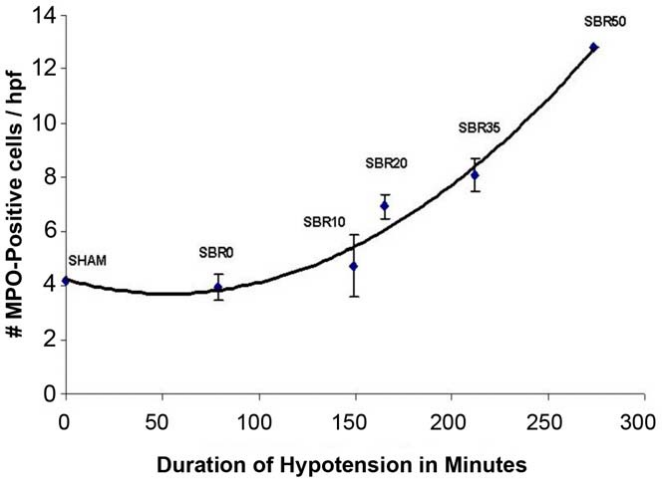
Effect of shock severity on liver inflammation. Rats were subjected to sham protocol (S) or to T/HS protocol with increasing duration of shock as indicated followed by resuscitation. The livers were harvested 60 minutes after the start of resuscitation. Sections of paraformaldehyde-fixed liver were stained to detect myeloperoxidase (MPO) activity and the number of MPO-positive cells counted. Data shown represents the number of MPO-positive cells/high power field in 20 random 1,000x fields. Curve fitting was performed and the best-fitting curve shown; the number of MPO-positive cells increased exponentially with duration of hypotension (Pearson correlation coefficient = 0.869, p<0.0001).

To determine the contribution of the severity of shock to liver inflammation, we assessed MPO-positive cells in the livers of rats subjected to increasing duration of shock. The number of MPO-positive cells/hpf increased with the duration of shock (Pearson correlation coefficient 0.869, p<0.0001), with the number of MPO-positive cells/hpf in the SBR50 group (12.8±0.09) increased 3.1 times over sham levels (4.2±0.01; p<0.001; Figure 1A).

**Figure 2:**
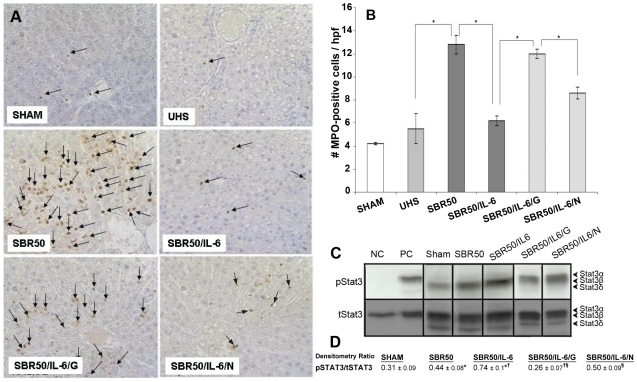
Effect of resuscitation, IL-6 treatment and GQ-ODN pre-treatment on T/HS-induced liver inflammation and Stat3 activation. Rats were subjected to sham protocol (Sham, n = 3), unresuscitated T/HS (UHS, n = 3), T/HS treated with placebo at the beginning of resuscitation (SBR50, n = 4), T/HS treated with IL-6 at the beginning of resuscitation (SBR50/IL-6, n = 4), T/HS preceded by treatment with GQ-oligodeoxynucleotide (GQ-ODN) 24 hours prior to resuscitation with IL-6 (SBR50/IL-6/G, n = 3), or T/HS preceded by treatment with nonspecific-ODN (NS-ODN) 24 hours prior to resuscitation with IL-6 (SBR50/IL-6N, n = 3). The livers were harvested 60 minutes after the start of resuscitation. MPO staining was performed in paraformaldehyde-fixed livers. Representative photomicrographs of 1000x fields of liver specimens from each experimental group are shown (2A). Arrows indicate MPO-positive cells. In Figure 2B, MPO-positive cells were counted in 20 hpf; data presented are the means ± SEM of each group (n≥5). Bars marked with an asterisk (*) differ significantly within the pair (p<0.05). Figure 2C shows representative immunoblots of protein extracts of whole liver from each group, a negative control (NC) cells and positive control (PC) cells developed with monoclonal antibodies to either phosphorylated (p)Stat3 and total (t)Stat3. Bands representing Stat3α, Stat3β, and Stat3δ isoforms are indicated on the right. Heavy lines separate representative samples from individual gels. Figure 2D shows the mean densitometric ratio of (p)Stat3 to (t)Stat3 bands ± standard deviation of the groups (n≥3 for each). Statistically significant (p<0.05) intergroup comparisons are indicated by “*”, “†”, and “§”, respectively.

To determine the contribution of resuscitation to liver inflammation, we assessed MPO positive cells/hpf in the livers of rats subjected to T/HS without resuscitation (UHS group) and compared these results with those obtained in the sham and the resuscitated SBR50 groups. The number of MPO-positive cells/hpf in the UHS group (5.5±1.3 cells/hpf) was 2.3 times lower than that of the SBR50 group (12.8±0.8 cells/hpf, p<0.01) and similar to that of the sham group (4.2±0.1 cells/hpf; Figures 2A and 2B). Thus, liver inflammation following T/HS depends on the severity of shock and requires resuscitation.

#### IL-6 administration at the beginning of resuscitation prevents T/HS-induced liver inflammation through a mechanism that involves Stat3α activation

To evaluate the effect of IL-6 administration on T/HS-induced liver inflammation, we measured MPO positive cells in the livers of rats subjected to T/HS with the most severe T/HS protocol (50% SBR) and randomly assigned to receive either PBS (SBR50) or IL-6 (10 µg/kg, SBR50/IL-6) at the beginning of resuscitation. The number of MPO-positive cells/hpf in the SBR50/IL-6 group (6.2±0.4) was 2.1-fold lower than that of the SBR50 group (12.8±0.8 cells/hpf, p<0.01) and similar to that of the sham group (4.2±0.1 cells/hpf; Figures 2A and 2B).

Stat3 is a member of the cytoplasmic protein family that is activated by a large number of extracellular stimuli including IL-6 [20]. Stat3 is known to activate expression of genes responsible for executing anti-inflammatory response, both *in vivo* and *in vitro* [21]. To assess if the anti-inflammatory effect of IL-6 in the liver is mediated by Stat3 activation, we determined if Stat3 is activated in the livers of rats resuscitated with IL-6. Extracts of cryotome sections of the liver harvested 1 hour after IL-6 treatment were examined by immunoblotting with mouse monoclonal antibody to Tyr705 phosphorylated (p)Stat3 (Figure 2C). Densitometric analysis of pStat3 bands normalized for total Stat3 indicated that Stat3 activity in livers of IL-6-treated rats (0.74±0.1 [SD]; n = 4) was increased 1.7 fold compared to the livers of placebo-treated rats (0.44±0.08 [SD]; n = 4; p<0.05, ANOVA; Figure 2D).

To confirm the role of Stat3 downstream of IL-6 in mediating its anti-inflammatory effect in the liver as well as to assess which isoform of Stat3 mediates it, we examined whether or not these effects of IL-6 could be reversed by pretreatment of rats with a G-rich oligodeoxynucleotide, G-quartet (GQ)-ODN, a novel Stat3 inhibitor, that forms a rigid G-quartet structure within cells, that inhibits the growth of tumors in which Stat3 is constitutively activated [22], [23]. Rats were treated in a blinded fashion with GQ-ODN (SBR50/IL-6/G group) or non-specific (NS) ODN (SBR50/IL-6/N group) 24 hours prior to being subjected to T/HS and resuscitation with IL-6 (Figure 2C). Densitometric analysis of pStat3 bands normalized for total Stat3 indicated that Stat3 activity in livers of SBR50/IL-6/G rats (0.26±0.07 [SD]; n = 3) was decreased 1.9 fold compared to in the livers of SBR50/IL-6/N rats (0.50±0.09 [SD]; n = 3; p<0.05, ANOVA; Figure 2D). Importantly, the inhibition of Stat3 activation within the livers of the SBR50/IL-6/G rats was accompanied by a return of the number of MPO-positive cells (12±0.4 cells/hpf) to levels similar to those of the placebo treated (SBR50) group (12.8±0.8 cells/hpf, p>0.05) and 2-fold higher than those of the IL-6 treated (SBR50/IL-6) group (6.2±0.4 cells/hpf, p<0.01; Figure 2A and 2B). Stat3 activity in livers of SBR50/IL-6/N rats (0.50±0.09 [SD]) was similar to that of the SBR50/IL-6 group (0.74±0.1 [SD]) and was accompanied by a number of MPO-positive cells/hpf that was indistinguishable from that of the SBR50/IL-6 group (Figure 2A and 2B). Thus, pharmacological inhibition of Stat3 using GQ-ODN in rats subjected to T/HS and resuscitated with IL-6 completely blocked IL-6-mediated Stat3 activation and IL-6-mediated prevention of liver inflammation.

**Figure 3:**
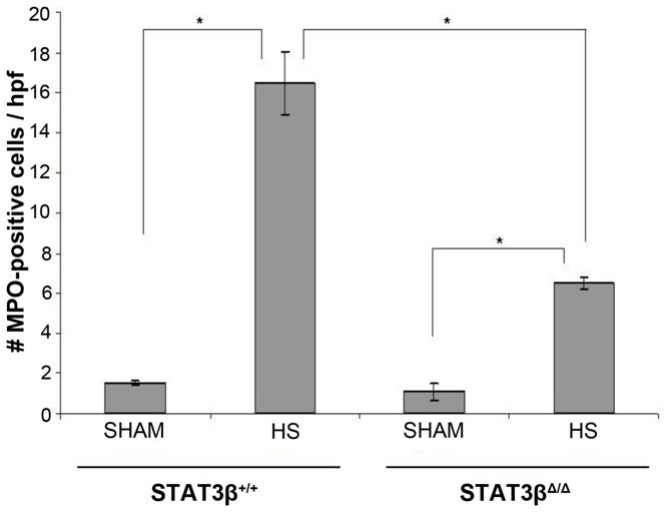
Effect of Stat3β ablation on T/HS-induced liver inflammation. Stat3β homozygous-deficient (Stat3β^Δ/Δ^) mice and their littermate control wild type mice were subjected to the murine T/HS protocol or sham protocol and their livers harvested 1 hr after the start of resuscitation. MPO-positive cells were counted in 20 HPF of paraformaldehyde-fixed liver. Data presented are the means ± SEM of each group (n≥3). Significant differences are indicated (Student's t-test).

Two isoforms of Stat3 are expressed in all cells, α(p92) and β(p83), both derived from a single gene by alternative mRNA splicing with Stat3α predominating. While mice deficient in both isoforms of Stat3 are embryonic lethal at day 6.5 to 7 [24] and mice deficient in Stat3α die within 24 hr of birth, mice deficient in Stat3β have normal survival and fertility [11]. To evaluate the hypothesis that Stat3, in particular Stat3α, contributes to IL-6-mediated prevention of liver inflammation and injury in the setting of T/HS, we subjected Stat3β homozygous-deficient (Stat3β^Δ/Δ^) mice and their littermate control wild type mice to our T/HS protocol (target MAP 30 mm Hg for 5 hr) and examined their livers for MPO-positive cells 1 hr after the start of resuscitation. Similar to our rat model, our mouse model of T/HS induced a 10-fold increase in the number of MPO positive cells/hpf in the wild type mice (16.5±0.79) compared to wild type mice subjected to sham protocol (1.6±0.16; p<0.001; Figure 3). While the number of MPO positive cells/hpf in the Stat3β^Δ/Δ^ mice subjected to T/HS (6.5±0.18) was higher than that of the Stat3β^Δ/Δ^ mice subjected to sham protocol (1.08±0.18; p<0.001), the number of MPO positive cells/hpf in the Stat3β^Δ/Δ^ mice subjected to T/HS was 2.5-fold lower than that of WT mice subjected to T/HS (p<0.001, Figure 3), indicating that the anti-inflammatory effects of Stat3 in the liver are mediated, at least in part, by Stat3α and that Stat3β inhibits the anti-inflammatory effects of Stat3α.

#### Microarray analysis of the liver inflammation transcriptome

In the context of normal wound healing and response to injury, Stat3 has been shown to repress transcription of pro-inflammatory genes, thereby promoting protection against necrosis and injury in organ such as the liver and lung [18], [25]–[27]. We have previously shown that IL-6 administration prevents T/HS-induced liver and lung inflammation, in part by blocking NF-κB activation and reducing pro-inflammatory cytokine production [28], [29]. To determine the mechanisms involved in this effect as well as to evaluate the role of Stat3 downstream of IL-6, globally, at the transcriptome level within the livers of animals subjected to T/HS especially those genes involved in inflammation in a global and unbiased manner, we performed Affymetrix oligonucleotide microarray analysis with RAE 230A chips. Fifteen chips were hybridized using mRNA isolated from 4 livers each from sham, SBR50, and SBR50/IL-6 groups, and 3 livers from SBR50/IL-6/G groups. All fifteen chips were included in the normalization and expression estimation steps of the analysis and were included in the statistical analysis and differential expression comparison. The 15,866 probesets on the RAE 230A chip represent 9,818 annotated genes or expressed sequence tags, including 694 inflammasome genes. The list of 694 genes belonging to the inflammasome, present on the RAE 230A (Table S1) was created by combining gene lists obtained by querying annotation databases provided in GeneSpring and dChip, which were derived from the Gene Ontology (GO) Consortium.

**Figure 4:**
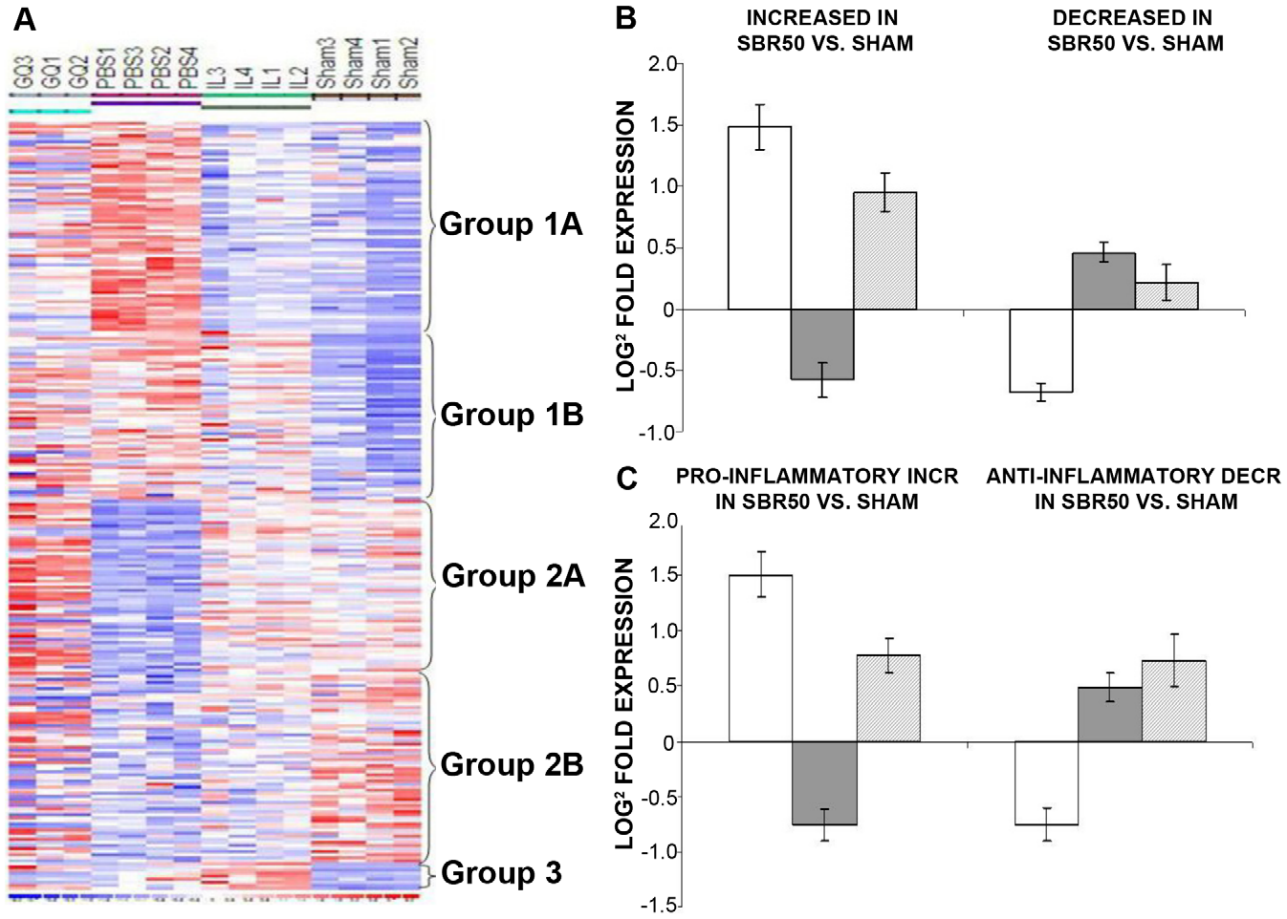
Effect of T/HS without or with IL-6 treatment on the liver inflammation transcriptome; impact of Stat3 inhibition on the IL-6 effect. In Figure 4A, a heat map of the liver inflammation transcriptome is shown containing those genes whose expression is altered within the 4 groups. Columns represent samples from the 4 groups examined as indicated (Sham; PBS, placebo-treated SBR50; IL, IL-6-treated SBR50/IL-6; and GQ, animals pre-treated with G-quartet ODN prior to HS and IL-6 treatment, SBR50/IL-6/G). Rows represent genes as listed in Table S2. Red indicates a level of expression above the mean expression of a gene within the experimental group. White indicates a level of expression at the mean within the experimental group while blue indicates a level of expression below the mean within the experimental groups. In Figure 4B, the 235 inflammation transcriptome genes whose expression levels were changed in SBR50 vs. sham were separated into those genes whose transcript levels were increased in SBR50 vs. sham (126 genes; left side of panel) and those whose transcript levels were decreased in SBR50 vs. sham (109 genes; right side of the panel). Bars shown represent mean ± SD of the Log_2_-fold change in gene expression levels for each comparison. In Figure 4C, the overall effect of T/HS in transcript levels of pro- and anti-inflammatory genes is shown. In the left side of the panel, the mean ± SD of the Log_2_-fold change in gene expression levels of 100 proinflammatory genes whose expression was increased in the SBR50 vs. sham comparison is shown (open bar). The expression of 84 of 100 of these genes was decreased in the SBR50/IL-6 vs. SBR50 comparison (gray bar). In the right side of the panel, the mean ± SD of the Log_2_-fold change in gene expression levels of 37 anti-inflammatory genes whose expression was decreased in the SBR50 vs. sham comparison is shown (open bar). The expression of 35 of these genes was increased in the SBR50/IL-6 vs. SBR50 comparison (gray bar).

To identify genes differentially expressed among the experimental groups, the data was filtered to remove genes with nearly uniformly low expression (absent on ≥80% of chips). Of the 694 inflammation transcriptome genes represented on the chips, 583 genes met the requirement of this filtering process and were included in the analysis (Table S1). One-way ANOVA (see Materials and Methods) was then performed which identified 352 inflammation transcriptome genes with differential expression among four experimental groups—sham, SBR50, SBR50/IL-6, and SBR50/IL-6/G—at a False Discovery Rate (FDR)  = 10% (Table S1). Of the 352 inflammation transcriptome genes whose expression was altered among the four groups, 235 were altered in the SBR50 vs. sham comparison (Table S2 and Figure 4A). Among the genes whose differential expression was altered in the SBR50 vs. sham comparison, the transcripts of the majority of these genes (126 genes) were increased in SBR50 vs. sham by 4.05±5.4 fold (range  = 1.07 to 45.41 fold) while transcripts of 109 genes were decreased in SBR50 vs. sham by 2.5±1.6 fold (range  = 1.1 to 5.3 fold; Figure 4B). Importantly, 104 of the 126 genes that were increased in the SBR50 vs. sham group, were decreased in the SBR50/IL-6 vs. SBR50 group by 1.8±0.9 fold (range  = 1.3 to 14.3 fold) and 103 of the 109 genes that were decreased in SBR50 group, were increased significantly in the SBR50/IL-6 group by 1.4±0.7 fold (range  = 1.2 to 2.9 fold; Figure 4B). Thus, of the 235 genes whose transcript levels were altered in SBR50 vs. sham group, 207 or 88% returned to sham level or were “normalized” in the SBR50/IL-6 group.

One-hundred and two of the 207 genes, which demonstrated altered expression in the SBR50 vs. sham comparison and normalization in the SBR50/IL-6 vs. SBR50 comparison, also demonstrated altered expression in the SBR50/IL-6 vs. SBR50/IL-6/G comparison. Ninety-seven of these 102 genes (95%) were altered in the opposite direction as the SBR50/IL-6 vs. SBR50 comparison, consistent with the hypothesis that IL-6 normalizes the T/HS-induced liver inflammation transcriptome alterations largely through activation of Stat3.

The inflammation transcriptome contains a list of genes encoding anti-inflammatory proteins that prevent inflammation and genes encoding proteins that induce inflammation. To identify candidate inflammation transcriptome genes whose altered expression mediates T/HS-induced liver inflammation and injury, we focused on both anti-inflammatory genes whose transcript levels were decreased by T/HS and on pro-inflammatory genes whose transcript levels were increased by T/HS. Among the genes differentially expressed in the SBR50 vs. sham comparison, 37 anti-inflammatory genes were decreased and 100 pro-inflammatory genes were increased (Figure 4C). Expression levels of 35 out of 37 (95%) anti-inflammatory genes decreased by T/HS were increased by IL-6 treatment; 84 of 100 (84%) of the pro-inflammatory genes that were increased by T/HS, were decreased by IL-6 treatment. Finally, the expression of 37% of anti-inflammatory genes increased by IL-6 treatment was decreased by pre-treatment with T40214; conversely 70% of pro-inflammatory genes decreased by IL-6 treatment were increased by T40214 pretreatment (Figure 4C).

Pro-inflammatory genes whose expression was increased≥6-fold by T/HS were *Dual specificity phosphatase 6* (*Dusp6*; 16.8-fold), *fatty acid binding protein 4 (Fabp4;* 12-fold*), alpha-2 macroglobulin (A2m,* 10.1-fold*), interferon regulatory factor 1* (*Irf1*; 9.3-fold), fatty *acid binding protein 5 (Fabp5;* 9.3-fold*), oxidized low density lipoprotein receptor 1* (*Oldlr1*; 9.0-fold), *Ephrin A1* (*Efna1;* 7.7-fold), *Phosphatidyl-inositol-3 kinase regulatory subunit polypeptide 1 (Pik3r1;* 6.2-fold) and *BCL2 adenovirus E1B interacting protein 3* (*Bnip3*; 6.1-fold, Table 1 ). The expression of each was decreased in the IL-6-treated group by 1.8- to 7.3-fold (Table S2). The effect of T/HS in the anti-inflammatory subset of genes was more modest. Indeed, none of the genes in this subset was altered more than 5.1-fold.

**Table 1:** Pro-inflammatory genes whose expression was increased ≥6-fold by T/HS.

Pro-inflammatory Gene Name	Symbol	Fold Change
*dual specificity phosphatase 6*	*Dusp6*	16.8
*fatty acid binding protein 4*	*Fabp4*	12
*alpha-2 macroglobulin*	*A2m*	10.1
*interferon regulatory factor 1*	*Irf1*	9.3
fatty *acid binding protein 5*	*Fabp5*	9.3
*oxidized low density lipoprotein receptor 1*	*Oldlr1*	9
*Ephrin A*	*Efna1*	7.7
*Phosphatidyl-inositol-3 kinase regulatory subunit polypeptide 1*	*Pik3r1*	6.2
*BCL2 adenovirus E1B interacting protein 3*	*Bnip3*	6.1

## Discussion

The findings in our rodent protocol of T/HS demonstrated that the extent of T/HS-induced liver inflammation following T/HS depends on the duration of hypotension and requires resuscitation. In previous studies, we had shown that liver inflammation induced by T/HS is associated with liver dysfunction and 72% mortality [12], [29], and IL-6 administration at the start of resuscitation completely reversed T/HS-induced liver inflammation and injury, decreasing mortality 5-fold, to 15% [12], [29]. In the current studies, we established that IL-6 administration at the start of resuscitation is capable of completely reversing liver inflammation as early as one hour after resuscitation, and is associated with increased liver Stat3 activation. Moreover, pharmacological inhibition of Stat3 using G-quartet oligodeoxynucleotide (GQ-ODN) T40214 developed by our group completely blocked IL-6-mediated liver Stat3 activation and prevention of inflammation. In our model of T/HS, Stat3α was the Stat3 isoform responsible for the anti-inflammatory effect of Stat3 in the liver. Liver microarray analysis showed that 65% of known inflammation transcriptome genes were altered in T/HS. Administration of IL-6 “normalized” the expression of 88% of these genes by increasing the expression levels of 95% of the anti-inflammatory gene transcripts whose levels were decreased by T/HS and by decreasing transcript levels of 84% of the pro-inflammatory genes whose levels were increased by T/HS. Furthermore, in 55% of the cases, Stat3 mediated the normalizing effect of IL-6 administration in the inflammation transcriptome.

The liver is readily susceptible to injury following insults such as hemorrhagic shock. Since the liver is responsible for maintaining homeostasis and is a key source of energy to other organs, hepatic injury and dysfunction associated with hemorrhagic shock can affect other organs and lead to multiple organ failure and death [30]–[32]. Liver inflammation and injury has been observed by us and others following T/HS [10], [29], [33]–[35], which we previously demonstrated can be prevented by administration of IL-6 at the start of resuscitation [28], [29]. However, the effect of the duration of hypotension on the development of T/HS-induced liver inflammation, as well as the mechanism(s) mediating the protective role of IL-6 administration have not been reported.

We have previously demonstrated that T/HS induces liver necrosis and polymorphonuclear (PMN) infiltration, which were observed 4 hrs after resuscitation [8], [29]. In the current study, using our rodent model of T/HS we found that liver inflammation occurs as early as 1 hour after reperfusion, and for the first time, that its severity depends on the duration of hypotension and requires resuscitation. Liver inflammation and injury have been shown to occur in settings of reperfusion injury such as liver transplantation [36], [37]. Upregulation of NF-κB target genes, which induce an inflammatory cascade, has been documented to occur early after hemorrhagic shock [28], [38]. Furthermore, NF-κB transcriptional activity has been shown to persist long after the initial insult, leading to a sustained inflammatory process, associated with cell injury [31]. Concordant with these data, a possible mechanism, mediating liver inflammation and its correlation with the severity of shock in our model of T/HS, invokes activation of NF-κB, which has been shown to be rapidly activated during reperfusion in hemorrhagic shock and whose binding activity depends on the severity of shock [39]. Activation of NF-κB can, in turn, upregulate the expression of cytokine genes that would promote inflammation [28], [29], [33]. The presence of nitric oxide, which has been implicated in the development of cell and organ damage in settings of regional hypoxia and low flow, both relevant to hemorrhagic shock [7], could also contribute to our findings of increased liver inflammation. Indeed, in previous studies, we demonstrated a nitric oxide-dependent increase of proinflammatory cytokines after HS [14], [33]. Reactive oxygen species (ROS) generated during reperfusion may also play a role in liver inflammation, as suggested by other investigators, given the ability of ROS to upregulate adhesion molecules and chemotactic factors which perpetuate the inflammatory response [7].

IL-6 is a pleiotropic cytokine, which has been shown to provide liver protection in various settings [40]-[43]. IL-6-mediated activation of Stat3 has been shown to down-regulate Stat1 activity and to prevent inflammation by blocking interferon-γ-like response *in vitro* [44]. Additionally, we have previously shown that exogenous IL-6 protected against liver PMN infiltration during hemorrhagic shock and was associated with decreased NF-κB activity in the liver 4 hrs after reperfusion [29]. Consistent with these findings, in the current study, we demonstrated that IL-6 prevented PMN infiltration as early as 1 hr after reperfusion. Likely explanations for our findings are the down-modulatory effect of IL-6 on NF-κB activity, either directly or as a result of reduced TNF-α or IL-1 production [29] and down-regulation of proinflammatory mediators by IL-6, which has been documented in T/HS animals treated with exogenous IL-6 infusion [28].

The role of Stat3 activation downstream of IL-6 in the resistance to T/HS-induced liver inflammation has not been studied previously. In our rodent model of T/HS we found increased Stat3 activation in the livers of rats subjected to T/HS and treated with IL-6 at the beginning of resuscitation. Furthermore, pharmacological inhibition of Stat3 with GQ-ODN, 40214, decreased liver Stat3 activation and completely prevented the anti-inflammatory effect of IL-6. Thus, IL-6-mediated activation of Stat3 is critical for protection against liver cell injury due to T/HS-induced inflammation.

Several studies have demonstrated that the protective role of Stat3 against liver injury is mediated, in part, by prevention of inflammation. Using a model of T-cell-mediated hepatitis in *IL-6^−/−^* mice, Hong et al. showed that IL-6-mediated Stat3 activity at the whole animal level protected the liver against inflammation [45]. Other groups, using a model of partial hepatectomy, have previously demonstrated that decreased activation of IL-6/Stat3 in the isolated hepatocytes of ethanol-fed rats was associated with increased liver inflammation and injury [46]. Furthermore, IL-6-induced activation of Stat3, assessed in total liver nuclear protein extract, has been shown to mediate liver protection against inflammation in a model of concavalin A liver injury using IL-6^−/−^ mice [47].

Similar to bowel inflammation [48]–[51], Stat3′s contribution to liver inflammation is influenced in a cell-type specific fashion that may functionally compete, depending on the cell type and the model of liver injury. Lafdil et al. recently demonstrated, in a model of T cell hepatitis, that deletion of Stat3 in myeloid cells enhances inflammation while deletion of Stat3 in T cells reduces liver inflammation [52]. Conditional deletion of Stat3 in hepatocytes has been shown to reduce liver inflammation while conditional deletion of Stat3 in myeloid cells enhanced liver inflammation after CCl4 injection [53]. We have previously demonstrated that the organ-sparing effect of IL-6 administration in our T/HS model is mediated by Stat3 activation within parenchymal cells, i.e. hepatocytes, cardiomyocytes, and alveolar epithelial cells of the liver, hear, and lung, respectively. While the anti-inflammatory effects of IL-6-activated Stat3 in our T/HS model may be mediated by Stat3 activation within myeloid cells, it is not likely to be mediated by Stat3 in T cells, and is most likely to be mediated by Stat3 activated within hepatocytes. However, future studies using the appropriate cell-specific Stat3 knockout animals will be necessary to distinguish between these alternatives.

Stat3 has multiple functions including modulation of target genes, which is in part due to the existence of two distinct isoforms, α (p92) and β (p83), derived from a single gene by alternative mRNA splicing with Stat3α predominating [54]. For example, Stat3α is essential for induction of inflammatory genes in the liver yet it also mediates the anti-inflammatory functions of IL-10 in macrophages [11]. In contrast, Stat3β^Δ/Δ^ mice are more susceptible to organ damage (particularly liver) and mortality due to lipopolysaccharide injection [11], [55]. Our finding of decreased MPO-positive cells in the livers of Stat3β^Δ/Δ^ mice subjected to T/HS shock in comparison to their wild type littermates, suggest that Stat3α, and not Stat3β, is the isoform mediating the anti-inflammatory effect of Stat3. The decrease in inflammmation is not mediated through differential levels of phosphorylated Stat3 within the isoform-depleted mice, but rather through the activation of distinct transcriptomes [11].

Similar to the prior discussion of Stat3′s varied role in inflammation depending on cell type, the role of Stat3α or Stat3β in inflammation is likely dependent on the target cell type as well as the pro-inflammatory stimulus. This is illustrated by the fact that Stat3α, not Stat3β, is known to mediate anti-inflammatory functions of IL-10 in LPS-stimulated macrophages [11], [56]. Additionally, Yu et al. found that in LPS-stimulated mesangial cells, Stat3α inhibited transcriptional activation of the inducible nitric oxide synthase (iNOS) gene by physically and functionally interacting with NF-κB [57]. Of note, we have previously shown that T/HS-induced inflammation and liver injury are mediated, in part, by iNOS transcription by NF-κB [58], [59].

Global and unbiased assessment of the liver inflammation transcriptome using oligonucleotide microarray analysis determined that T/HS altered the expression of 65% of inflammation transcriptome genes, of which 73% were pro-inflammatory and 27% were anti-inflammatory genes (S2, Figure 4B). T/HS increased the levels of 58% of proinflammatory genes and decreased the levels of 59% of anti-inflammatory genes. IL-6 administration prevented the T/HS-mediated changes by decreasing the expression of inflammation transcriptome genes whose expression was increased by T/HS, and by increasing the expression of inflammation transcriptome genes whose expression was decreased by T/HS (Figure 4C), indicating that IL-6 administration had a “normalizing” effect on the T/HS-induced liver inflammation transcriptome. Inhibition of Stat3 using GQ-ODN T40214 reversed the IL-6 “normalizing” effect on gene expression in 55% of the inflammation transcriptome genes (Figure 4C). These results suggest that IL-6 administration prevents T/HS-induced liver inflammation by opposing the effects of T/HS on the liver inflammation transcriptome, in part, by Stat3 activation.

Anti- and proinflammatory subsets of genes were analyzed separately to determine the effect of T/HS in each subset of transcripts. Gene transcript levels of the majority of proinflammatory were increased by T/HS. Interestingly, IL-6 normalized the expression of 84% of these genes, an effect that was reversed by pre-treatment with Stat3 inhibitor in the majority of them (66%, Table S2, Figure 4C). In our model of T/HS, proinflammatory genes upregulated by T/HS are likely to be mediators of liver inflammation, which is likely prevented by IL-6 normalizing effect on their expression through Stat3 activation. Among the transcripts most upregulated following T/HS and downregulated in the IL-6 treatment group were *Dusp6, Fabp4, A2m, Irf1, Fabp5, Oldlr1, Efna1, Pik3r1,* and *Bnip3.* Dusp6 is a cytosolic phosphatase with specifically inactivates extracellular signal-regulated kinase (ERK) [60]. *Oldlr1* is expressed in endothelial cells and activated macrophages. Oldlr1 is a type II glycoprotein and acts as a receptor for oxidized low-density lipoprotein (ox-LDL). Interaction with ox-LDL induces ROS, reduces NO and activates NF-κB. *Old1r*1 is known to induce endothelial cell injury by facilitating inflammatory cell adhesion in an animal model of myocardial ischemia-reperfusion [61], [62]. *Fabp2* and *Fabp5* have been implicated in the pathogenesis of allergic airway inflammation as well as chronic inflammation associated with cardiovascular disease [63], [64]. *A2m* is a major acute phase protein with protease-inhibitory activity, and mice deficient in *A2m* were protected against lethal systemic inflammation induced by TNF [65]. Activation of *Irf1*-dependent autocrine loop is known to induce persistent inflammatory response in an animal model of delayed inflammation [66].

Mediation of the protective effects of IL-6 in T/HS-induced liver injury is in large part through Stat3′s ability to prevent liver inflammation. Our findings provide evidence that support the use of IL-6 as a potential therapeutic agent to protect against liver injury and dysfunction by blocking inflammation early after reperfusion. Such an intervention may prevent multiple organ failure and improve survival in the setting of trauma complicated by severe hemorrhagic shock.

## Supporting Information

Table S1 **Inflammation transcriptome genes examined in the microarray experiments.** Table S1 identifies the members of the inflammasome present on the RAE 230A chip after filtering for uniformly low expression across chips and describes the 352 identified as having differential expression among four experimental groups—Sham, SBR50, SBR50/IL-6, and SBR50/IL-6/G—at a False Discovery Rate (FDR)  =  10% via Oneway ANOVA (see Methods section). * “Y” indicates signal detected above background for gene probeset in 20% or more of the chips. ^†^ “Y” indicates significant differential gene expression within the Sham, SBR50, SBR50/IL-6, and SBR50/IL-6/G groups using False Discovery Rate (FDR)  =  10%. “N” indicates not significant differential gene expression. “NA” indicates genes not included in the analysis because of not being detected in at least 20% of the chips.
(DOC)

Table S2 **Inflammation transcriptome genes differentially expressed in the SBR50 vs. SHAM comparison.** Table S2 identifies the 235 members of the inflammasome whose expression was altered among the experimental groups and describes the pattern of dysregulation between and among experimental groups in comparison to the SBR50 vs. Sham animals. Genes are grouped based on direction of dysregulation, allowing for identification of inflammasome genes dysregulated by our trauma/hemorrhagic shock model that are “normalized” by IL-6 and identification of inflammasome genes whose altered expression is, in part, regulated through Stat3. Group 1A represents genes increased in SBR50 vs. Sham and decreased in SBR50/IL-6 vs. SBR50. Group 1B represents genes increased in SBR50 vs. Sham and unchanged in SBR50/IL-6 vs. SBR50. Group 2A presents genes decreased in SBR50 vs. Sham and increased in SBR50/IL-6 vs. SBR50. Group 2B presents genes decreased in SBR50 vs. Sham and unchanged in SBR50/IL-6 vs. SBR50. Group 3 represents genes increased in SBR50 vs. Sham and increased in SBR50/IL-6 vs. SBR50. ^*^Genes listed in regular type are anti-inflammatory, while genes listed in italics are pro-inflammatory. ^†^FDR: False discovery rate.
(DOC)
