# Supplementary material for: IL-6-Mediated Activation of Stat3α Prevents Trauma/Hemorrhagic Shock-Induced Liver Inflammation
Source: PLoS One. 2011 Jun 29;6(6):e21449. doi: 10.1371/journal.pone.0021449 (PMC3127578; doi:10.1371/journal.pone.0021449)
Supplement: Table S1 — Inflammation transcriptome genes examined in the microarray experiments. Table S1 identifies the members of the inflammasome present on the RAE 230A chip after filtering for uniformly low expression across chips and describes the 352 identified as having differential expression among four experimental groups—Sham, SBR50, SBR50/IL-6, and SBR50/IL-6/G—at a False Discovery Rate (FDR) = 10% via Oneway ANOVA (see Methods section). * “Y” indicates signal detected above background for gene probeset in 20% or more of the chips. † “Y” indicates significant differential gene expression within the Sham, SBR50, SBR50/IL-6, and SBR50/IL-6/G groups using False Discovery Rate (FDR) = 10%. “N” indicates not significant differential gene expression. “NA” indicates genes not included in the analysis because of not being detected in at least 20% of the chips. (DOC) [file pone.0021449.s001.doc]

**Table S1**. Inflammation transcriptome genes examined in the microarray experiments.

| # | **GENE ID** | **GENE NAME** | **PRESENT*** | **SIGN†** |
| --- | --- | --- | --- | --- |
| 1 | Z18877 | 2',5'-oligoadenylate synthetase 1, 40/46kDa | Y | N |
| 2 | BF417479 | 24-dehydrocholesterol reductase | Y | Y |
| 3 | AF068268 | 2-5 oligoadenylate synthetase 1B | Y | N |
| 4 | BF419319 | 2'-5' oligoadenylate synthetase-like 1 | Y | N |
| 5 | AI177093 | 5-azacytidine induced gene 2 | Y | N |
| 6 | NM_017250 | 5-hydroxytryptamine (serotonin) receptor 2B | Y | N |
| 7 | NM_020308 | a disintegrin and metallopeptidase domain 15 (metargidin) | Y | N |
| 8 | BI300565 | a disintegrin and metalloprotease domain 10 | Y | Y |
| 9 | AI013474 | abhydrolase domain containing 2 (predicted) | Y | N |
| 10 | BI296498 | Activated leukocyte cell adhesion molecule | Y | N |
| 11 | NM_130756 | acyl-CoA thioesterase 8 | Y | Y |
| 12 | NM_053669 | adaptor protein with pleckstrin homology and src homology 2 domains | Y | Y |
| 13 | BE113312 | adaptor-related protein complex 3, beta 1 subunit (predicted) | Y | Y |
| 14 | BI282757 | adaptor-related protein complex 3, delta 1 subunit | Y | N |
| 15 | M69045 | adenosine A1 receptor | Y | N |
| 16 | AF228684 | adenosine A2a receptor | Y | Y |
| 17 | NM_017161 | adenosine A2B receptor | Y | Y |
| 18 | NM_012896 | adenosine A3 receptor | Y | N |
| 19 | NM_130399 | adenosine deaminase | Y | N |
| 20 | AI237657 | ADP-ribosylation factor-like 6 interacting protein 2 | Y | Y |
| 21 | AY057895 | adrenergic receptor, beta 2 | Y | N |
| 22 | NM_012715 | Adrenomedullin | Y | Y |
| 23 | BI284400 | ajuba homolog (Xenopus laevis) | Y | Y |
| 24 | NM_134326 | Albumin | Y | N |
| 25 | NM_019363 | aldehyde oxidase 1 | Y | Y |
| 26 | NM_017196 | allograft inflammatory factor 1 | Y | N |
| 27 | NM_012901 | alpha 1 microglobulin/bikunin | Y | N |
| 28 | NM_023103 | alpha-1-inhibitor III /// Murinoglobulin 1 homolog (mouse) | Y | Y |
| 29 | NM_012826 | alpha-2-glycoprotein 1, zinc | Y | Y |
| 30 | NM_012898 | alpha-2-HS-glycoprotein | Y | N |
| 31 | NM_012488 | alpha-2-macroglobulin | Y | Y |
| 32 | AB039823 | alpha-2u globulin PGCL3 // similar to alpha-2u-globulin | Y | N |
| 33 | NM_012493 | alpha-fetoprotein | Y | Y |
| 34 | AI070137 | amine oxidase, copper containing 3 | Y | N |
| 35 | BI274184 | amyotrophic lateral sclerosis 2 (juvenile) homolog (human) | Y | N |
| 36 | NM_134432 | angiotensinogen (serpin peptidase inhibitor, clade A, member 8) | Y | Y |
| 37 | L81174 | ankyrin repeat domain 1 (cardiac muscle) | Y | N |
| 38 | NM_012904 | annexin A1 | Y | Y |
| 39 | BF287964 | annexin A11 | Y | N |
| 40 | NM_019905 | annexin A2 | Y | Y |
| 41 | NM_013132 | annexin A5 | Y | N |
| 42 | NM_031612 | apelin, AGTRL1 ligand | Y | N |
| 43 | NM_013112 | apolipoprotein A-II | Y | N |
| 44 | J02582 | apolipoprotein E | Y | N |
| 45 | AI180413 | apolipoprotein H | Y | Y |
| 46 | BF284937 | apolipoprotein L, 3 /// similar to RIKEN cDNA 2210421G13 | Y | N |
| 47 | BE116857 | apoptotic chromatin condensation inducer 1 | Y | Y |
| 48 | AF218388 | apoptotic peptidase activating factor 1 | Y | N |
| 49 | NM_022960 | aquaporin 9 | Y | N |
| 50 | NM_012822 | arachidonate 5-lipoxygenase | Y | N |
| 51 | NM_017260 | arachidonate 5-lipoxygenase activating protein | Y | Y |
| 52 | NM_017134 | arginase 1 | Y | N |
| 53 | BI282724 | arginine vasopressin-induced 1 | Y | Y |
| 54 | BG378089 | ash2 (absent, small, or homeotic)-like (Drosophila) (predicted) | Y | N |
| 55 | NM_131907 | ATPase, Ca++-sequestering | Y | Y |
| 56 | BF416408 | ATPase, H+ transporting, lysosomal V0 subunit a isoform 2 (predicted) | Y | Y |
| 57 | BG380494 | ATP-binding cassette, sub-family F (GCN20), member 1 | Y | N |
| 58 | AB038388 | Attractin | Y | Y |
| 59 | NM_031328 | B-cell CLL/lymphoma 10 | Y | N |
| 60 | NM_016993 | B-cell leukemia/lymphoma 2 | Y | N |
| 61 | AI172204 | B-cell receptor-associated protein 29 | Y | N |
| 62 | AI409930 | B-cell receptor-associated protein 31 | Y | Y |
| 63 | NM_053420 | BCL2/adenovirus E1B 19 kDa-interacting protein 3 | Y | Y |
| 64 | NM_080888 | BCL2/adenovirus E1B 19 kDa-interacting protein 3-like | Y | Y |
| 65 | AF279911 | bcl2-associated death promoter | Y | N |
| 66 | AF235993 | Bcl2-associated X protein | Y | Y |
| 67 | NM_053739 | beclin 1 (coiled-coil, myosin-like BCL2-interacting protein) | Y | Y |
| 68 | AW916647 | Beta-2 microglobulin | Y | N |
| 69 | NM_017300 | bile acid-Coenzyme A: amino acid N-acyltransferase | Y | Y |
| 70 | NM_030851 | bradykinin receptor B1 | Y | N |
| 71 | X69681 | bradykinin receptor, beta 2 | Y | N |
| 72 | NM_012931 | breast cancer anti-estrogen resistance 1 | Y | Y |
| 73 | NM_053303 | Burkitt lymphoma receptor 1 | Y | N |
| 74 | NM_053670 | calcitonin gene-related peptide-receptor component protein | Y | N |
| 75 | NM_017338 | calcitonin/calcitonin-related polypeptide, alpha | Y | N |
| 76 | H35646 | calcium activated nucleotidase 1 | Y | N |
| 77 | BE099992 | calcium/calmodulin-dependent protein kinase kinase 2, beta | Y | N |
| 78 | AI408948 | carbonic anhydrase 2 | Y | Y |
| 79 | BF281311 | casein kinase 2, beta subunit | Y | N |
| 80 | BF556820 | Casitas B-lineage lymphoma b | Y | N |
| 81 | D85899 | caspase 1 | Y | N |
| 82 | BM387008 | caspase 3, apoptosis related cysteine protease | Y | Y |
| 83 | NM_053736 | caspase 4, apoptosis-related cysteine peptidase | Y | N |
| 84 | NM_022277 | caspase 8 | Y | Y |
| 85 | AI102738 | Catenin (cadherin associated protein), beta 1 | Y | N |
| 86 | NM_022597 | cathepsin B | Y | N |
| 87 | NM_017097 | cathepsin C | Y | Y |
| 88 | NM_012938 | cathepsin E | Y | N |
| 89 | NM_017320 | cathepsin S | Y | N |
| 90 | NM_012524 | CCAAT/enhancer binding protein (C/EBP), alpha | Y | N |
| 91 | NM_024125 | CCAAT/enhancer binding protein (C/EBP), beta | Y | N |
| 92 | NM_013087 | CD 81 antigen | Y | Y |
| 93 | NM_021744 | CD14 antigen | Y | Y |
| 94 | NM_031812 | CD164 antigen | Y | N |
| 95 | NM_017079 | CD1d1 antigen | Y | Y |
| 96 | BG378032 | CD2 antigen (cytoplasmic tail) binding protein 2 (predicted) | Y | Y |
| 97 | BI285141 | CD24 antigen | Y | Y |
| 98 | NM_022259 | CD244 natural killer cell receptor 2B4 | Y | Y |
| 99 | BF398424 | CD276 antigen | Y | N |
| 100 | NM_013169 | CD3 antigen delta polypeptide | Y | N |
| 101 | AA945909 | CD3 antigen, epsilon polypeptide (predicted) | Y | N |
| 102 | AI044631 | CD3 antigen, gamma polypeptide | Y | N |
| 103 | L08447 | CD3 antigen, zeta polypeptide | Y | N |
| 104 | NM_031561 | cd36 antigen /// similar to fatty acid translocase/CD36 (predicted) | Y | N |
| 105 | NM_012705 | CD4 antigen | Y | N |
| 106 | X13016 | CD48 antigen | Y | N |
| 107 | NM_019295 | CD5 antigen | Y | N |
| 108 | NM_012523 | CD53 antigen | Y | Y |
| 109 | NM_012925 | CD59 antigen | Y | N |
| 110 | NM_013069 | CD74 antigen (invariant polypeptide of major histocompatibility complex, class II antigen-associated) | Y | N |
| 111 | AI412355 | CD83 antigen (predicted) | Y | Y |
| 112 | NM_020081 | cd86 antigen | Y | N |
| 113 | AI227627 | CD9 antigen | Y | Y |
| 114 | BI296525 | CD97 antigen | Y | N |
| 115 | AI599324 | CDC42 effector protein (Rho GTPase binding) 5 (predicted) | Y | N |
| 116 | NM_019296 | cell division cycle 2 homolog A (S. pombe) | Y | N |
| 117 | NM_019205 | chemokine (C-C motif) ligand 11 | Y | N |
| 118 | NM_031530 | chemokine (C-C motif) ligand 2 | Y | N |
| 119 | AF053312 | chemokine (C-C motif) ligand 20 | Y | N |
| 120 | AF163477 | chemokine (C-C motif) ligand 22 | Y | N |
| 121 | AI058901 | chemokine (C-C motif) ligand 27 (predicted) | Y | N |
| 122 | U22414 | chemokine (C-C motif) ligand 3 | Y | N |
| 123 | U06434 | chemokine (C-C motif) ligand 4 | Y | N |
| 124 | NM_031116 | chemokine (C-C motif) ligand 5 | Y | N |
| 125 | BE095824 | chemokine (C-C motif) ligand 6 | Y | N |
| 126 | NM_020542 | chemokine (C-C motif) receptor 1 | Y | Y |
| 127 | NM_021866 | chemokine (C-C motif) receptor 2 | Y | N |
| 128 | NM_133532 | chemokine (C-C motif) receptor 4 | Y | N |
| 129 | NM_053960 | chemokine (C-C motif) receptor 5 | Y | N |
| 130 | NM_133534 | chemokine (C-X3-C) receptor 1 | Y | N |
| 131 | NM_030845 | chemokine (C-X-C motif) ligand 1 | Y | Y |
| 132 | U22520 | chemokine (C-X-C motif) ligand 10 | Y | N |
| 133 | AF189724 | chemokine (C-X-C motif) ligand 12 | Y | Y |
| 134 | BG380414 | chemokine (C-X-C motif) ligand 14 | Y | Y |
| 135 | NM_053647 | chemokine (C-X-C motif) ligand 2 | Y | N |
| 136 | AI169104 | chemokine (C-X-C motif) ligand 4 | Y | Y |
| 137 | NM_022214 | chemokine (C-X-C motif) ligand 5 | Y | Y |
| 138 | AF349115 | chemokine (C-X-C motif) ligand 7 | Y | N |
| 139 | AI170387 | chemokine (C-X-C motif) ligand 9 | Y | N |
| 140 | AA945737 | chemokine (C-X-C motif) receptor 4 | Y | Y |
| 141 | NM_078621 | chemokine binding protein 2 | Y | N |
| 142 | AF253064 | chemokine-like factor | Y | N |
| 143 | NM_033233 | chorionic somatomammotropin hormone 1-like 1 | Y | N |
| 144 | NM_013166 | ciliary neurotrophic factor | Y | N |
| 145 | NM_053529 | class II, major histocompatibility complex, transactivator | Y | N |
| 146 | AI717113 | coagulation factor 5 (mapped) | Y | N |
| 147 | NM_022924 | coagulation factor II | Y | N |
| 148 | NM_012950 | coagulation factor II (thrombin) receptor | Y | N |
| 149 | NM_053897 | coagulation factor II (thrombin) receptor-like 1 | Y | N |
| 150 | AF310076 | coagulation factor II (thrombin) receptor-like 2 | Y | N |
| 151 | NM_013057 | coagulation factor III | Y | N |
| 152 | NM_017143 | coagulation factor X | Y | N |
| 153 | BM388525 | Coagulation factor XIII, A1 subunit | Y | N |
| 154 | BI284441 | collectin sub-family member 12 | Y | N |
| 155 | BI285793 | colony stimulating factor 1 receptor | Y | N |
| 156 | NM_017104 | colony stimulating factor 3 (granulocyte) | Y | Y |
| 157 | NM_019259 | complement component 1, q subcomponent binding protein | Y | N |
| 158 | BF418957 | complement component 1, q subcomponent, alpha polypeptide | Y | N |
| 159 | AW434057 | complement component 1, q subcomponent, beta polypeptide | Y | N |
| 160 | AI411618 | complement component 1, q subcomponent, gamma polypeptide | Y | N |
| 161 | AF136537 | complement component 1, q subcomponent, receptor 1 | Y | N |
| 162 | D88250 | complement component 1, s subcomponent /// similar to complement component 1, s subcomponent (predicted) | Y | Y |
| 163 | NM_016994 | complement component 3 | Y | Y |
| 164 | NM_012516 | complement component 4 binding protein, alpha | Y | Y |
| 165 | NM_016995 | complement component 4 binding protein, beta | Y | N |
| 166 | BI285347 | complement component 4a /// complement component 4, gene 2 | Y | Y |
| 167 | NM_053619 | complement component 5, receptor 1 | Y | Y |
| 168 | AA819870 | complement component 8, beta polypeptide (mapped) | Y | Y |
| 169 | NM_057146 | complement component 9 | Y | Y |
| 170 | NM_130409 | complement component factor H | Y | N |
| 171 | AI639117 | complement factor B | Y | Y |
| 172 | AI237358 | complement factor D (adipsin) | Y | N |
| 173 | NM_024157 | complement factor I | Y | Y |
| 174 | D42115 | complement receptor related protein | Y | N |
| 175 | NM_022266 | connective tissue growth factor | Y | N |
| 176 | AI598397 | cornichon homolog (Drosophila) (predicted) | Y | N |
| 177 | NM_130411 | coronin, actin binding protein 1A | Y | N |
| 178 | NM_017096 | C-reactive protein, pentraxin-related | Y | N |
| 179 | NM_022253 | Csk binding protein | Y | N |
| 180 | AI105444 | CXXC finger 5 | Y | N |
| 181 | X64589 | cyclin B1 | Y | N |
| 182 | NM_012766 | cyclin D3 | Y | Y |
| 183 | NM_031030 | cyclin G associated kinase | Y | Y |
| 184 | AI010427 | cyclin-dependent kinase inhibitor 1A | Y | Y |
| 185 | NM_022501 | cysteine-rich protein 2 | Y | Y |
| 186 | NM_053641 | cysteinyl leukotriene receptor 1 | Y | N |
| 187 | U39208 | cytochrome P450 4F6 | Y | Y |
| 188 | U90271 | cytotoxic T-lymphocyte-associated protein 4 | Y | N |
| 189 | NM_024131 | D-dopachrome tautomerase | Y | Y |
| 190 | NM_022849 | deleted in malignant brain tumors 1 | Y | Y |
| 191 | AF178975 | deoxyribonuclease II | Y | N |
| 192 | NM_012789 | dipeptidylpeptidase 4 | Y | Y |
| 193 | NM_022934 | DnaJ (Hsp40) homolog, subfamily A, member 1 | Y | Y |
| 194 | NM_032079 | DnaJ (Hsp40) homolog, subfamily A, member 2 | Y | N |
| 195 | BI282224 | DnaJ (Hsp40) homolog, subfamily A, member 3 | Y | Y |
| 196 | AI104324 | DnaJ (Hsp40) homolog, subfamily A, member 4 | Y | Y |
| 197 | BM384926 | DnaJ (Hsp40) homolog, subfamily B, member 1 (predicted) | Y | Y |
| 198 | BI295873 | DnaJ (Hsp40) homolog, subfamily B, member 11 | Y | N |
| 199 | AI175031 | DnaJ (Hsp40) homolog, subfamily B, member 4 | Y | N |
| 200 | NM_012699 | DnaJ (Hsp40) homolog, subfamily B, member 9 | Y | Y |
| 201 | NM_053690 | DnaJ (Hsp40) homolog, subfamily C, member 14 | Y | Y |
| 202 | BM390416 | DnaJ (Hsp40) homolog, subfamily C, member 15 (predicted) | Y | N |
| 203 | AA964764 | DnaJ (Hsp40) homolog, subfamily C, member 2 | Y | N |
| 204 | NM_022232 | DnaJ (Hsp40) homolog, subfamily C, member 3 | Y | Y |
| 205 | BG380252 | DnaJ (Hsp40) homolog, subfamily C, member 4 | Y | N |
| 206 | U39320 | DnaJ (Hsp40) homolog, subfamily C, member 5 | Y | N |
| 207 | BF392285 | DnaJ (Hsp40) homolog, subfamily C, member 7 | Y | N |
| 208 | AI227785 | DnaJ (Hsp40) homolog, subfamily C, member 8 | Y | Y |
| 209 | AI138041 | dnaj-like protein | Y | N |
| 210 | BI288838 | docking protein 4 (predicted) | Y | N |
| 211 | L12407 | dopamine beta hydroxylase | Y | N |
| 212 | NM_031352 | drebrin-like | Y | Y |
| 213 | BE111118 | dual specificity phosphatase 19 (predicted) | Y | N |
| 214 | AI408580 | dual specificity phosphatase 2 | Y | N |
| 215 | AI172067 | dual specificity phosphatase 22 (predicted) | Y | N |
| 216 | NM_053883 | dual specificity phosphatase 6 | Y | Y |
| 217 | AF329091 | E-3 epididymal fluid protein | Y | N |
| 218 | AA818055 | E74-like factor 1 | Y | N |
| 219 | NM_012551 | early growth response 1 | Y | Y |
| 220 | BI288690 | ectonucleoside triphosphate diphosphohydrolase 2 | Y | N |
| 221 | BF404078 | Ena-vasodilator stimulated phosphoprotein | Y | N |
| 222 | NM_053936 | endothelial differentiation, lysophosphatidic acid G-protein-coupled receptor, 2 | Y | N |
| 223 | NM_017192 | endothelial differentiation, sphingolipid G-protein-coupled receptor, 5 | Y | Y |
| 224 | NM_053599 | ephrin A1 | Y | Y |
| 225 | NM_017089 | ephrin B1 | Y | N |
| 226 | NM_012842 | epidermal growth factor | Y | Y |
| 227 | NM_021689 | epiregulin | Y | N |
| 228 | NM_022936 | epoxide hydrolase 2, cytoplasmic | Y | N |
| 229 | BI298225 | exosome component 9 | Y | N |
| 230 | M69056 | farnesyltransferase, CAAX box, beta | Y | N |
| 231 | NM_080895 | Fas apoptotic inhibitory molecule | Y | N |
| 232 | NM_012908 | Fas ligand (TNF superfamily, member 6) | Y | N |
| 233 | NM_130406 | Fas-associated factor 1 | Y | Y |
| 234 | NM_053365 | fatty acid binding protein 4, adipocyte | Y | Y |
| 235 | U13253 | fatty acid binding protein 5, epidermal | Y | Y |
| 236 | BF415939 | FBJ murine osteosarcoma viral oncogene homolog | Y | Y |
| 237 | AI011757 | Fc fragment of IgG, low affinity IIIa, receptor | Y | N |
| 238 | NM_053843 | Fc gamma receptor II beta | Y | Y |
| 239 | M17153 | Fc receptor, IgE, high affinity I, alpha polypeptide | Y | N |
| 240 | BE110597 | Fc receptor, IgG, alpha chain transporter | Y | N |
| 241 | X73371 | Fc receptor, IgG, low affinity IIb | Y | Y |
| 242 | NM_053843 | Fc receptor, IgG, low affinity III /// Fc gamma receptor II beta | Y | Y |
| 243 | NM_012848 | ferritin, heavy polypeptide 1 | Y | N |
| 244 | AA875097 | fibrinogen, alpha polypeptide | Y | Y |
| 245 | M35602 | fibrinogen, B beta polypeptide | Y | N |
| 246 | NM_012559 | fibrinogen, gamma polypeptide | Y | Y |
| 247 | NM_019305 | fibroblast growth factor 2 | Y | N |
| 248 | S54008 | Fibroblast growth factor receptor 1 | Y | N |
| 249 | NM_053429 | fibroblast growth factor receptor 3 | Y | N |
| 250 | AA893484 | fibronectin 1 | Y | Y |
| 251 | BE113154 | FK506 binding protein 4 | Y | N |
| 252 | H35236 | Fms interacting protein | Y | N |
| 253 | AI231684 | forkhead box O3a (predicted) | Y | N |
| 254 | NM_012953 | fos-like antigen 1 | Y | Y |
| 255 | AI230396 | fyn proto-oncogene | Y | N |
| 256 | NM_012755 | fyn proto-oncogene | Y | N |
| 257 | AF038388 | FYVE, RhoGEF and PH domain containing 4 | Y | N |
| 258 | NM_053969 | G protein pathway suppressor 1 | Y | N |
| 259 | BI294756 | G protein pathway suppressor 2 (predicted) | Y | N |
| 260 | BI275972 | G protein-coupled receptor 89 (predicted) | Y | Y |
| 261 | L22654 | gamma-2a immunoglobulin heavy chain /// similar to gamma-2a immunoglobulin heavy chain | Y | N |
| 262 | AW520967 | gamma-aminobutyric acid (GABA-A) receptor, subunit alpha 5 | Y | N |
| 263 | NM_031756 | gamma-glutamyl carboxylase | Y | Y |
| 264 | NM_019235 | gamma-glutamyltransferase-like activity 1 | Y | Y |
| 265 | AI411352 | gap junction membrane channel protein alpha 1 | Y | Y |
| 266 | D21095 | gene model 1960, (NCBI) | Y | N |
| 267 | NM_017009 | glial fibrillary acidic protein | Y | N |
| 268 | NM_017165 | glutathione peroxidase 4 | Y | N |
| 269 | NM_012795 | glycoprotein 5, platelet | Y | N |
| 270 | BI273727 | G-protein signalling modulator 3 (AGS3-like, C. elegans) | Y | N |
| 271 | BI287978 | growth arrest and DNA-damage-inducible 45 beta | Y | Y |
| 272 | AI599423 | growth arrest and DNA-damage-inducible 45 gamma | Y | Y |
| 273 | X62853 | growth factor receptor bound protein 2 | Y | N |
| 274 | BI291231 | GTP binding protein 1 (predicted) | Y | N |
| 275 | NM_031034 | guanine nucleotide binding protein, alpha 12 | Y | N |
| 276 | AW143805 | Guanine nucleotide binding protein, alpha q polypeptide | Y | N |
| 277 | NM_024138 | guanine nucleotide binding protein, gamma 7 | Y | Y |
| 278 | NM_013170 | guanylate cyclase 2C | Y | Y |
| 279 | NM_133624 | guanylate nucleotide binding protein 2 | Y | N |
| 280 | NM_012582 | haptoglobin | Y | N |
| 281 | NM_053612 | heat shock 22kDa protein 8 | Y | Y |
| 282 | NM_031971 | heat shock 70kD protein 1A /// heat shock 70kD protein 1B (mapped) | Y | Y |
| 283 | AI172496 | heat shock transcription factor 1 | Y | N |
| 284 | NM_024385 | hematopoietically expressed homeobox | Y | Y |
| 285 | NM_012580 | heme oxygenase (decycling) 1 | Y | Y |
| 286 | AF176534 | hemochromatosis | Y | Y |
| 287 | NM_017017 | hepatocyte growth factor | Y | N |
| 288 | NM_022180 | hepatocyte nuclear factor 4, alpha | Y | Y |
| 289 | NM_053469 | hepcidin antimicrobial peptide | Y | Y |
| 290 | AI103467 | heterogeneous nuclear ribonucleoprotein M | Y | Y |
| 291 | NM_024363 | heterogeneous nuclear ribonucleoproteins methyltransferase-like 2 (S. cerevisiae) | Y | Y |
| 292 | BF419085 | histone deacetylase 4 (predicted) | Y | N |
| 293 | AI103918 | histone deacetylase 7A | Y | N |
| 294 | BM392321 | homeodomain interacting protein kinase 2 (predicted) | Y | Y |
| 295 | NM_013179 | hypocretin | Y | N |
| 296 | BM390522 | hypothetical gene supported by NM_130426 | Y | N |
| 297 | BM392257 | Hypoxanthine guanine phosphoribosyl transferase | Y | N |
| 298 | BE109152 | IK cytokine | Y | N |
| 299 | AI176519 | immediate early response 3 | Y | Y |
| 300 | NM_031624 | immunoglobulin (CD79A) binding protein 1 | Y | N |
| 301 | AJ391299 | immunoglobulin heavy chain (alpha polypeptide) (mapped)//similar to immunoglobulin heavy chain variable region (predicted) | Y | N |
| 302 | AI411947 | immunoglobulin heavy chain 1a (serum IgG2a) | Y | N |
| 303 | NM_133542 | immunoglobulin superfamily, member 6 | Y | N |
| 304 | NM_023973 | indoleamine-pyrrole 2,3 dioxygenase | Y | N |
| 305 | NM_022610 | inducible T-cell co-stimulator | Y | N |
| 306 | NM_012590 | inhibin alpha | Y | N |
| 307 | NM_017128 | inhibin beta-A | Y | N |
| 308 | NM_013060 | inhibitor of DNA binding 2 | Y | Y |
| 309 | AF000942 | inhibitor of DNA binding 3 | Y | N |
| 310 | NM_080899 | inhibitor of kappa light polypeptide enhancer in B-cells, kinase complex-associated protein | Y | N |
| 311 | NM_053355 | inhibitor of kappaB kinase beta | Y | N |
| 312 | BF281278 | inosine 5-monophosphate dehydrogenase 2 | Y | Y |
| 313 | NM_019311 | inositol polyphosphate-5-phosphatase D | Y | N |
| 314 | NM_019129 | insulin 1 | Y | N |
| 315 | NM_013144 | insulin-like growth factor binding protein 1 | Y | Y |
| 316 | BE108969 | insulin-like growth factor binding protein 4 | Y | N |
| 317 | NM_030994 | integrin alpha 1 | Y | Y |
| 318 | NM_012967 | intercellular adhesion molecule 1 | Y | Y |
| 319 | AI410467 | intercellular adhesion molecule 2 | Y | Y |
| 320 | AA900618 | interferon (alpha and beta) receptor 1 (predicted) | Y | N |
| 321 | AF010466 | interferon gamma | Y | N |
| 322 | BM389261 | interferon gamma inducible protein 30 | Y | Y |
| 323 | NM_053783 | interferon gamma receptor 1 | Y | Y |
| 324 | AI602715 | interferon gamma receptor 2 (predicted) | Y | N |
| 325 | NM_030833 | interferon induced transmembrane protein 2 (1-8D) | Y | Y |
| 326 | NM_012591 | interferon regulatory factor 1 | Y | Y |
| 327 | BF411036 | interferon regulatory factor 7 | Y | N |
| 328 | BF284262 | Interferon regulatory factor 8 | Y | N |
| 329 | BM388891 | interferon-induced protein 35 | Y | Y |
| 330 | NM_017019 | interleukin 1 alpha | Y | N |
| 331 | NM_031512 | interleukin 1 beta | Y | N |
| 332 | NM_012968 | interleukin 1 receptor accessory protein | Y | N |
| 333 | NM_022194 | interleukin 1 receptor antagonist | Y | Y |
| 334 | NM_013123 | interleukin 1 receptor, type I | Y | N |
| 335 | L02926 | interleukin 10 | Y | N |
| 336 | NM117539 | Interleukin 10 receptor, alpha | N | NA |
| 337 | NM304091 | Interleukin 10 receptor, beta | N | NA |
| 338 | AF347936 | interleukin 11 receptor, alpha chain 1 | Y | Y |
| 339 | NM84405 | Interleukin 12a | Y | N |
| 340 | NM64546 | Interleukin 12b | N | NA |
| 341 | NM053828 | Interleukin 13 | N | NA |
| 342 | NM145789 | Interleukin 13 receptor, alpha 1 | N | NA |
| 343 | NM171060 | Interleukin 13 receptor, alpha 2 | N | NA |
| 344 | AF015718 | interleukin 15 | Y | N |
| 345 | NM116996 | Interleukin 16 | N | NA |
| 346 | NC005115 | Interleukin 17 receptor, B | N | NA |
| 347 | AJ222813 | interleukin 18 | Y | N |
| 348 | AI178808 | interleukin 2 receptor, gamma (severe combined immunodeficiency) | Y | Y |
| 349 | NM155140 | Interleukin 23 receptor alpha, subunit p19 | N | NA |
| 350 | NM_031513 | interleukin 3 | Y | N |
| 351 | NM_133380 | interleukin 4 receptor, alpha | Y | Y |
| 352 | NM_012589 | interleukin 6 | Y | N |
| 353 | NM_017020 | interleukin 6 receptor, alpha | Y | N |
| 354 | AA851740 | Interleukin 6 signal transducer | Y | N |
| 355 | AF367210 | interleukin 7 | Y | N |
| 356 | NM54258 | Interleukin 8 receptor, alpha | Y | N |
| 357 | NM29385 | Interleukin 8 receptor, beta | N | NA |
| 358 | NM116558 | Interleukin 9 receptor | Y | N |
| 359 | NM24500 | Interleukin 9 receptor | N | NA |
| 360 | NM_019128 | internexin, alpha | Y | N |
| 361 | AB062135 | J domain protein 1 | Y | N |
| 362 | NM_019147 | jagged 1 | Y | N |
| 363 | BI274746 | jagged 2 | Y | N |
| 364 | NM_031514 | Janus kinase 2 | Y | Y |
| 365 | NM_012855 | Janus kinase 3 | Y | N |
| 366 | NM_012725 | kallikrein B, plasma 1 | Y | N |
| 367 | U56824 | killer cell lectin-like receptor, subfamily A, member 5 /// Ly49 stimulatory receptor 7 /// Ly49s8 | Y | N |
| 368 | NM_012745 | killer cell lectin-like receptor, subfamily D, member 1 | Y | N |
| 369 | U93307 | kinase insert domain protein receptor | Y | Y |
| 370 | NM_012696 | kininogen 1 /// K-kininogen /// similar to alpha-1 major acute phase protein prepeptide | Y | Y |
| 371 | NM_012741 | K-kininogen | Y | Y |
| 372 | NM_031135 | Kruppel-like factor 10 | Y | N |
| 373 | NM_031642 | Kruppel-like factor 6 | Y | Y |
| 374 | NM_012976 | lectin, galactose binding, soluble 5 /// lectin, galactose binding, soluble 9 | Y | N |
| 375 | U72741 | lectin, galactose binding, soluble 9 | Y | N |
| 376 | NM_053886 | lectin, mannose-binding, 1 | Y | Y |
| 377 | NM_030856 | leucine rich repeat protein 3, neuronal | Y | N |
| 378 | NM_022634 | leucocyte specific transcript 1 | Y | N |
| 379 | NM_022196 | leukemia inhibitory factor | Y | N |
| 380 | AW918009 | leukotriene A4 hydrolase | Y | N |
| 381 | NM_030853 | linker for activation of T cells | Y | N |
| 382 | NM_031621 | linker of T-cell receptor pathways | Y | Y |
| 383 | BF289368 | lipopolysaccharide binding protein | Y | N |
| 384 | BI284739 | LPS-induced TN factor | Y | Y |
| 385 | AW434178 | lymphocyte antigen 6 complex, locus H (predicted) | Y | N |
| 386 | AA874924 | lymphocyte antigen 86 (predicted) | Y | N |
| 387 | BE109711 | lymphocyte cytosolic protein 1 | Y | N |
| 388 | NM_130421 | lymphocyte cytosolic protein 2 | Y | N |
| 389 | AI137137 | lymphocyte protein tyrosine kinase (mapped) | Y | N |
| 390 | AI012109 | lymphocyte specific 1 | Y | N |
| 391 | NM_080769 | lymphotoxin A | Y | N |
| 392 | AI705656 | lymphotoxin B receptor | Y | Y |
| 393 | L12458 | lysozyme | Y | Y |
| 394 | NM_031051 | macrophage migration inhibitory factor | Y | N |
| 395 | NM_024352 | Macrophage stimulating 1 (hepatocyte growth factor-like) | Y | Y |
| 396 | NM_013130 | MAD homolog 1 (Drosophila) | Y | N |
| 397 | BI301490 | major histocompatibility complex, class II, DM alpha | Y | N |
| 398 | AI171966 | major histocompatibility complex, class II, DM beta | Y | N |
| 399 | BE111083 | mannan-binding lectin serine peptidase 1 | Y | Y |
| 400 | AA996755 | mannan-binding lectin serine peptidase 2 | Y | Y |
| 401 | NM_012599 | mannose binding lectin 1, protein A | Y | Y |
| 402 | NM_022704 | mannose binding lectin 2 (protein C) | Y | Y |
| 403 | NM_012845 | membrane-spanning 4-domains, subfamily A, member 2 | Y | Y |
| 404 | NM_031517 | met proto-oncogene | Y | Y |
| 405 | AF024712 | MHC class Ib antigen (predicted) | Y | N |
| 406 | BI290559 | microsomal glutathione S-transferase 2 (predicted) | Y | Y |
| 407 | AA892234 | microsomal glutathione S-transferase 3 (predicted) | Y | N |
| 408 | NM_030859 | midkine | Y | N |
| 409 | NM_053842 | mitogen activated protein kinase 1 | Y | N |
| 410 | NM_031020 | mitogen activated protein kinase 14 | Y | N |
| 411 | AF155236 | mitogen activated protein kinase 3 | Y | N |
| 412 | NM_053777 | mitogen activated protein kinase 8 interacting protein | Y | Y |
| 413 | BI283843 | mitogen activated protein kinase kinase 3 | Y | Y |
| 414 | NM_017246 | mitogen activated protein kinase kinase 5 | Y | N |
| 415 | NM_053887 | mitogen activated protein kinase kinase kinase 1 | Y | Y |
| 416 | NM_013055 | mitogen activated protein kinase kinase kinase 12 | Y | N |
| 417 | AA925300 | mitogen activated protein kinase kinase kinase 3 (predicted) | Y | Y |
| 418 | AI146037 | Mitogen activated protein kinase kinase kinase 7 (predicted) | Y | N |
| 419 | NM_017322 | mitogen-activated protein kinase 9 | Y | Y |
| 420 | NM_053703 | mitogen-activated protein kinase kinase 6 | Y | N |
| 421 | BE107454 | mitogen-activated protein kinase kinase kinase 7 interacting protein 2 | Y | N |
| 422 | AA819812 | mitogen-activated protein kinase kinase kinase kinase 3 | Y | Y |
| 423 | BG372713 | Monoglyceride lipase | Y | Y |
| 424 | M22359 | Murinoglobulin 1 homolog (mouse) | Y | Y |
| 425 | AW251450 | musculoskeletal, embryonic nuclear protein 1 | Y | N |
| 426 | NM_017026 | myelin basic protein | Y | N |
| 427 | AI236590 | myeloid differentiation primary response gene 88 | Y | Y |
| 428 | BG381670 | myeloid leukemia factor 2 (predicted) | Y | Y |
| 429 | BI296048 | myeloid-associated differentiation marker | Y | Y |
| 430 | X52711 | myxovirus (influenza virus) resistance 1 | Y | N |
| 431 | NM_017028 | myxovirus (influenza virus) resistance 2 | Y | N |
| 432 | AA945624 | NAD(P)H dehydrogenase, quinone 2 | Y | Y |
| 433 | AA894045 | natural cytotoxicity triggering receptor 3 | Y | N |
| 434 | NM_024361 | N-deacetylase/N-sulfotransferase (heparan glucosaminyl) 1 | Y | N |
| 435 | BG671512 | Nedd4 family interacting protein 1 | Y | N |
| 436 | U02315 | neuregulin 1 | Y | Y |
| 437 | BE107450 | Neuronal regeneration related protein | Y | Y |
| 438 | L14447 | neurotrophic tyrosine kinase, receptor, type 3 | Y | N |
| 439 | NM_053734 | neutrophil cytosolic factor 1 | Y | N |
| 440 | AF218575 | nibrin | Y | N |
| 441 | U72660 | ninjurin 1 | Y | Y |
| 442 | NC005109 | Nitric oxide synthase 1, adaptor protein (neuronal) | N | NA |
| 443 | NC005103 | Nitric oxide synthase 1, neuronal | Y | N |
| 444 | NM012611 | Nitric oxide synthase 2, inducible | N | NA |
| 445 | NM021838 | Nitric oxide synthase 3, endothelial cell | N | NA |
| 446 | BM384099 | N-myc downstream regulated gene 1 | Y | Y |
| 447 | BM386507 | non-catalytic region of tyrosine kinase adaptor protein 1 (predicted) | Y | N |
| 448 | AA957410 | non-catalytic region of tyrosine kinase adaptor protein 2 (predicted) | Y | Y |
| 449 | AI011448 | Notch gene homolog 2 (Drosophila) | Y | Y |
| 450 | AI599986 | Notch homolog 4 | Y | N |
| 451 | BG377358 | nuclear factor of activated T-cells, cytoplasmic, calcineurin-dependent 4 | Y | N |
| 452 | AA858801 | nuclear factor of kappa light chain gene enhancer in B-cells 1, p105 | Y | N |
| 453 | AW672589 | nuclear factor of kappa light chain gene enhancer in B-cells inhibitor, alpha | Y | Y |
| 454 | AI170362 | nuclear factor of kappa light polypeptide gene enhancer in B-cells 2, p49/p100 | Y | N |
| 455 | AI179123 | nuclear factor, erythroid derived 2,-like 1 (predicted) | Y | N |
| 456 | NM_053727 | nuclear factor, interleukin 3 regulated | Y | Y |
| 457 | AF228043 | nuclear receptor coactivator 6 | Y | N |
| 458 | AY066016 | nuclear receptor subfamily 3, group C, member 1 | Y | Y |
| 459 | U72345 | nuclear receptor subfamily 4, group A, member 2 | Y | N |
| 460 | BI286040 | nucleoporin 62 | Y | N |
| 461 | NM_012617 | opioid receptor, delta 1 | Y | N |
| 462 | L22536 | opioid receptor, kappa 1 | Y | N |
| 463 | L20684 | opioid receptor, mu 1 | Y | N |
| 464 | NM_053288 | orosomucoid 1 | Y | N |
| 465 | NM_130402 | osteoclast inhibitory lectin | Y | N |
| 466 | NM_133306 | oxidized low density lipoprotein (lectin-like) receptor 1 | Y | Y |
| 467 | NM_053289 | pancreatitis-associated protein | Y | N |
| 468 | NM_031975 | parathymosin | Y | Y |
| 469 | BG673589 | paxillin | Y | Y |
| 470 | NM_053373 | peptidoglycan recognition protein 1 | Y | N |
| 471 | NM_017330 | perforin 1 (pore forming protein) | Y | N |
| 472 | BI282076 | peroxiredoxin 4 | Y | N |
| 473 | AI230294 | Peroxisome proliferator activated receptor delta | Y | N |
| 474 | NM_013124 | peroxisome proliferator activated receptor gamma | Y | N |
| 475 | NM_057137 | phenylalkylamine Ca2+ antagonist (emopamil) binding protein | Y | N |
| 476 | NM_053481 | phosphatidylinositol 3-kinase, catalytic, beta polypeptide | Y | Y |
| 477 | D64048 | phosphatidylinositol 3-kinase, regulatory subunit, polypeptide 1 | Y | Y |
| 478 | NM_053866 | phospholipase A2, activating protein | Y | Y |
| 479 | NM_053758 | phospholipase C, epsilon 1 | Y | Y |
| 480 | NM_057194 | phospholipid scramblase 1 | Y | Y |
| 481 | NM_031640 | plasma glutamate carboxypeptidase | Y | Y |
| 482 | NM_053491 | plasminogen | Y | Y |
| 483 | NM_013151 | plasminogen activator, tissue | Y | Y |
| 484 | AF007789 | plasminogen activator, urokinase receptor | Y | Y |
| 485 | AI010414 | platelet/endothelial cell adhesion molecule | Y | N |
| 486 | NM_053321 | platelet-activating factor receptor | Y | N |
| 487 | AI178056 | pleckstrin homology domain containing, family G (with RhoGef domain) member 5 | Y | N |
| 488 | AB020726 | podocalyxin-like | Y | N |
| 489 | NM_031821 | polo-like kinase 2 (Drosophila) | Y | N |
| 490 | AB019366 | poly (ADP-ribose) glycohydrolase | Y | Y |
| 491 | BM383696 | poly A binding protein, cytoplasmic 4 | Y | Y |
| 492 | NM_023021 | potassium intermediate/small conductance calcium-activated channel, subfamily N, member 4 | Y | N |
| 493 | U05989 | PRKC, apoptosis, WT1, regulator | Y | Y |
| 494 | NM_012630 | prolactin receptor | Y | N |
| 495 | AI411541 | Prostaglandin E receptor 3 (subtype EP3) | Y | Y |
| 496 | NM_032076 | prostaglandin E receptor 4 (subtype EP4) | Y | N |
| 497 | AF280967 | prostaglandin E synthase | Y | N |
| 498 | U03389 | prostaglandin-endoperoxide synthase 2 | Y | N |
| 499 | NM_017264 | proteasome (prosome, macropain) 28 subunit, alpha | Y | Y |
| 500 | NM_017257 | proteasome (prosome, macropain) 28 subunit, beta | Y | Y |
| 501 | BG373505 | proteasome (prosome, macropain) subunit, beta type 10 | Y | N |
| 502 | NM_012803 | protein C | Y | Y |
| 503 | AI137406 | protein C receptor, endothelial | Y | Y |
| 504 | BE108748 | protein inhibitor of activated STAT, 4 | Y | N |
| 505 | BF415343 | protein kinase C, alpha | Y | N |
| 506 | NM_133307 | protein kinase C, delta | Y | Y |
| 507 | NM_017175 | protein kinase N1 | Y | N |
| 508 | NM_019142 | protein kinase, AMP-activated, alpha 1 catalytic subunit | Y | Y |
| 509 | AI102009 | protein kinase, interferon inducible double stranded RNA dependent activator | Y | N |
| 510 | NM_022951 | protein phosphatase 1, regulatory subunit 10 | Y | N |
| 511 | NM_017038 | protein phosphatase 1A, magnesium dependent, alpha isoform | Y | Y |
| 512 | AI233712 | protein phosphatase 1D magnesium-dependent, delta isoform (predicted) | Y | N |
| 513 | NM_017039 | protein phosphatase 2 (formerly 2A), catalytic subunit, alpha isoform | Y | Y |
| 514 | NM_017040 | protein phosphatase 2 (formerly 2A), catalytic subunit, beta isoform | Y | Y |
| 515 | AI411788 | protein phosphatase 2 (formerly 2A), regulatory subunit A (PR 65), beta isoform | Y | Y |
| 516 | NM_017042 | protein phosphatase 3, catalytic subunit, beta isoform | Y | N |
| 517 | NM_021701 | protein phosphatase 3, regulatory subunit B, alpha isoform (calcineurin B, type II) | Y | N |
| 518 | NM_031729 | protein phosphatase 5, catalytic subunit | Y | N |
| 519 | U06230 | protein S (alpha) | Y | Y |
| 520 | AI172465 | Protein tyrosine phosphatase, non-receptor type 11 | Y | N |
| 521 | NM_053908 | protein tyrosine phosphatase, non-receptor type 6 | Y | N |
| 522 | NM_013016 | protein tyrosine phosphatase, non-receptor type substrate 1 | Y | N |
| 523 | M10072 | protein tyrosine phosphatase, receptor type, C | Y | N |
| 524 | NM_080767 | proteosome (prosome, macropain) subunit, beta type 8 | Y | Y |
| 525 | AI599350 | proteosome (prosome, macropain) subunit, beta type 9 | Y | N |
| 526 | X92069 | purinergic receptor P2X, ligand-gated ion channel, 5 | Y | N |
| 527 | U76206 | purinergic receptor P2Y, G-protein coupled, 14 | Y | Y |
| 528 | BI282953 | PYD and CARD domain containing | Y | N |
| 529 | NM_017317 | RAB27A, member RAS oncogene family | Y | N |
| 530 | M83681 | RAB3D, member RAS oncogene family | Y | N |
| 531 | BF407276 | Rap guanine nucleotide exchange factor (GEF) 2 (predicted) | Y | Y |
| 532 | NM_130824 | RAS guanyl releasing protein 4 | Y | N |
| 533 | AI408053 | ras homolog gene family, member A | Y | N |
| 534 | AA891940 | ras homolog gene family, member C (predicted) | Y | N |
| 535 | AF036537 | receptor-interacting serine-threonine kinase 3 | Y | N |
| 536 | L20869 | regenerating islet-derived 3 gamma | Y | N |
| 537 | BM386789 | regulator of G-protein signaling 1 | Y | N |
| 538 | U27767 | regulator of G-protein signaling 4 | Y | Y |
| 539 | BF419009 | Regulator of G-protein signalling 3 | Y | N |
| 540 | AI111991 | regulatory factor X, 1 (influences HLA class II expression) (predicted) | Y | N |
| 541 | AJ299017 | ret proto-oncogene | Y | N |
| 542 | AI408677 | Rho GDP dissociation inhibitor (GDI) alpha | Y | N |
| 543 | BF285771 | Rho, GDP dissociation inhibitor (GDI) beta | Y | N |
| 544 | BG668512 | ribosomal protein S19 | Y | Y |
| 545 | AJ005023 | RT1 class I, A3 | Y | N |
| 546 | M24026 | RT1 class I, CE12 | Y | N |
| 547 | U50449 | RT1 class I, CE16 | Y | N |
| 548 | BI300597 | RT1 class I, CE5 | Y | N |
| 549 | BM389027 | RT1 class I, locus Ke4 | Y | N |
| 550 | L23128 | RT1 class Ib gene, H2-TL-like, grc region (N1) /// RT1 class Ib gene, H2-TL-like, grc region (N3) /// RT1 class Ib gene, H2-TL-like, grc region(N2) | Y | N |
| 551 | AI500830 | RT1 class Ib, locus Aw2 | Y | N |
| 552 | BI395698 | RT1 class Ib, locus Aw2 /// histocompatibility 2, T region locus 24 /// RT1 class I, CE2 /// RT1-149 protein /// RT1 class I, CE15 /// RT1 class I, CE10 | Y | N |
| 553 | M64795 | RT1 class Ib, locus Aw2 /// RT1 class I, CE12 /// RT1 class I, CE1 /// RT1 class I, CE2 /// RT1 class I, CE15 | Y | N |
| 554 | AJ276126 | RT1 class Ib, locus Aw2 /// RT1 class I, CE5 | Y | N |
| 555 | AJ249701 | RT1 class Ib, locus Aw2 /// RT1 class Ia, locus A2 /// RT1 class I, A3 | Y | N |
| 556 | NM_022921 | RT1 class Ib, locus M3 | Y | N |
| 557 | AJ243974 | RT1 class Ib, locus S3 | Y | Y |
| 558 | BG378249 | RT1 class II, locus Ba | Y | N |
| 559 | AI715202 | RT1 class II, locus Bb | Y | N |
| 560 | Y00480 | RT1 class II, locus Da | Y | N |
| 561 | BI279526 | RT1 class II, locus Db1 | Y | N |
| 562 | BI282965 | RT1-149 protein | Y | N |
| 563 | NM_053822 | S100 calcium binding protein A8 (calgranulin A) | Y | Y |
| 564 | NM_053587 | S100 calcium binding protein A9 (calgranulin B) | Y | Y |
| 565 | NM_013191 | S100 protein, beta polypeptide | Y | N |
| 566 | NM_053687 | schlafen 3 | Y | N |
| 567 | AI009823 | secreted and transmembrane 1 | Y | N |
| 568 | AB001382 | secreted phosphoprotein 1 | Y | Y |
| 569 | BI296054 | selectin, platelet | Y | N |
| 570 | AI411586 | sequestosome 1 | Y | Y |
| 571 | NM_022957 | serine (or cysteine) peptidase inhibitor, clade A, member 5 | Y | Y |
| 572 | NM_024382 | serine (or cysteine) peptidase inhibitor, clade D, member 1 | Y | Y |
| 573 | NM_012620 | serine (or cysteine) peptidase inhibitor, clade E, member 1 | Y | Y |
| 574 | AW915763 | serine (or cysteine) peptidase inhibitor, clade G, member 1 | Y | N |
| 575 | NM_022519 | serine (or cysteine) proteinase inhibitor, clade A (alpha-1 antiproteinase, antitrypsin), member 1 | Y | N |
| 576 | NM_017170 | serum amyloid P-component | Y | N |
| 577 | NM_021676 | SH3/ankyrin domain gene 3 /// hypothetical gene supported by NM_021676 | Y | Y |
| 578 | NM_054011 | SH3-domain binding protein 5 (BTK-associated) | Y | Y |
| 579 | BF550890 | sialophorin | Y | N |
| 580 | AA819349 | sideroflexin 1 | Y | Y |
| 581 | NM_032612 | signal transducer and activator of transcription 1 | Y | Y |
| 582 | AA799569 | Signal transducer and activator of transcription 2 | Y | Y |
| 583 | BE113920 | signal transducer and activator of transcription 3 | Y | Y |
| 584 | NM_017064 | signal transducer and activator of transcription 5A | Y | N |
| 585 | AI177626 | signal transducer and activator of transcription 5B | Y | N |
| 586 | BG663097 | signal transducer and activator of transcription interacting protein 1 | Y | Y |
| 587 | BI282085 | similar to 1700029B21Rik protein | Y | N |
| 588 | AI012393 | similar to butyrophilin-like 8 (predicted) /// butyrophilin-like 7 /// butyrophilin-like 8 | Y | N |
| 589 | AF244895 | similar to DnaJ (Hsp40) homolog, subfamily B, member 12 | Y | Y |
| 590 | AA818949 | similar to DnaJ (Hsp40) homolog, subfamily B, member 12 /// similar to DnaJ (Hsp40) homolog, subfamily B, member 12 | Y | Y |
| 591 | AI411693 | similar to immunoglobulin heavy chain 6 (Igh-6) /// similar to Ig H-chain V-region precursor /// similar to single chain Fv antibody fragment scFv 7-10A /// similar to Ig heavy chain V region MC101 precursor | Y | Y |
| 592 | AI029631 | similar to immunoglobulin light chain variable region (predicted) | Y | N |
| 593 | BM388282 | Similar to KIAA0962 protein | Y | Y |
| 594 | AI071180 | similar to KIAA1086 protein (predicted) | Y | N |
| 595 | BE104797 | Similar to Liver-expressed antimicrobial peptide 2 precursor (LEAP-2) | Y | N |
| 596 | BF283018 | similar to RIKEN cDNA 2810451A06 | Y | Y |
| 597 | BE099060 | similar to RIKEN cDNA D430028G21 | Y | N |
| 598 | AA892854 | similar to Small inducible cytokine B13 precursor (CXCL13) (B lymphocyte chemoattractant) (CXC chemokine BLC) | Y | N |
| 599 | BI288244 | small inducible cytokine subfamily E, member 1 | Y | N |
| 600 | X89383 | SNF related kinase | Y | N |
| 601 | BI293600 | Solute carrier family 35, member B2 | Y | Y |
| 602 | M97656 | somatostatin receptor 1 | Y | N |
| 603 | BI293504 | SP140 nuclear body protein | Y | N |
| 604 | BI289536 | spastic paraplegia 21 homolog (human) | Y | Y |
| 605 | AF217088 | sperm associated antigen 11 | Y | N |
| 606 | U21683 | spleen tyrosine kinase | Y | N |
| 607 | AA801238 | spondin 2, extracellular matrix protein | Y | N |
| 608 | AI409218 | Sprouty-related, EVH1 domain containing 2 | Y | N |
| 609 | NM_130413 | src family associated phosphoprotein 2 | Y | N |
| 610 | BI280327 | STIP1 homology and U-Box containing protein 1 | Y | N |
| 611 | NM_017050 | superoxide dismutase 1 | Y | Y |
| 612 | BG671549 | superoxide dismutase 2, mitochondrial | Y | Y |
| 613 | BG377057 | suppression of tumorigenicity 13 | Y | Y |
| 614 | BE100794 | suppressor of cytokine signaling 1 | Y | N |
| 615 | NM_053565 | suppressor of cytokine signaling 3 | Y | Y |
| 616 | AI170864 | suppressor of cytokine signaling 6 (predicted) | Y | N |
| 617 | BG671061 | tachykinin 1 | Y | N |
| 618 | NM_033098 | TAP binding protein | Y | N |
| 619 | AW919577 | T-cell receptor beta chain | Y | N |
| 620 | Z27087 | T-cell receptor gamma chain | Y | N |
| 621 | AI406496 | tec protein tyrosine kinase | Y | N |
| 622 | NM_133526 | tetraspanin 8 | Y | N |
| 623 | BG381669 | tetratricopeptide repeat domain 7 | Y | Y |
| 624 | NM_022584 | thioredoxin reductase 2 | Y | N |
| 625 | BG666306 | thrombomodulin | Y | Y |
| 626 | NM_031133 | thrombopoietin | Y | N |
| 627 | NM_012687 | thromboxane A synthase 1 | Y | N |
| 628 | BE111601 | Thymoma viral proto-oncogene 1 | Y | N |
| 629 | AI105076 | thymoma viral proto-oncogene 2 | Y | N |
| 630 | AI145313 | thymus cell antigen 1, theta | Y | N |
| 631 | AI101391 | Tial1 cytotoxic granule-associated RNA binding protein-like 1 (mapped) | Y | N |
| 632 | NM_017200 | tissue factor pathway inhibitor | Y | Y |
| 633 | AI179507 | tissue factor pathway inhibitor 2 | Y | Y |
| 634 | NM_053819 | tissue inhibitor of metalloproteinase 1 | Y | Y |
| 635 | NM_021989 | tissue inhibitor of metalloproteinase 2 | Y | Y |
| 636 | AW919111 | TNFAIP3 interacting protein 1 (predicted) | Y | N |
| 637 | AA957069 | Toll interacting protein (predicted) | Y | N |
| 638 | NM310553 | Toll-like receptor 2 | Y | N |
| 639 | AF057025 | toll-like receptor 4 | Y | N |
| 640 | NM317468 | Toll-like receptor 7 | N | NA |
| 641 | NM684440 | Toll-like receptor 8 | N | NA |
| 642 | NM338457 | Toll-like receptor 9 | N | NA |
| 643 | BE095882 | toll-like receptor adaptor molecule 2 (predicted) | Y | N |
| 644 | AW140505 | TRAF family member-associated Nf-kappa B activator /// hypothetical gene supported by NM_145788 | Y | N |
| 645 | BM388871 | transcription elongation factor A (SII) 1 /// similar to transcription elongation factor IIS - mouse | Y | N |
| 646 | AW252817 | transcription elongation factor A (SII), 3 | Y | Y |
| 647 | AI231686 | Transcription elongation factor B (SIII), polypeptide 3 | Y | Y |
| 648 | BG381660 | transcription factor 8 | Y | N |
| 649 | BI284455 | transcription factor Pur-beta | Y | N |
| 650 | NM_012671 | transforming growth factor alpha | Y | N |
| 651 | BF420705 | Transforming growth factor, beta 2 | Y | N |
| 652 | AI556917 | transforming, acidic coiled-coil containing protein 3 | Y | N |
| 653 | NM_053785 | transmembrane 4 superfamily member 4 | Y | Y |
| 654 | X57523 | transporter 1, ATP-binding cassette, sub-family B (MDR/TAP) | Y | N |
| 655 | NM_032056 | transporter 2, ATP-binding cassette, sub-family B (MDR/TAP) | Y | N |
| 656 | NM_013042 | trefoil factor 3 | Y | N |
| 657 | BI287875 | Trk-fused gene | Y | N |
| 658 | BI282847 | Tryptophan hydroxylase 1 | Y | Y |
| 659 | U24150 | tuberous sclerosis 2 | Y | Y |
| 660 | NM_057149 | tumor necrosis factor (ligand) superfamily, member 11 | Y | N |
| 661 | AA800814 | tumor necrosis factor (ligand) superfamily, member 13 | Y | N |
| 662 | AA819227 | tumor necrosis factor (TNF superfamily, member 2) | Y | N |
| 663 | BF283688 | tumor necrosis factor ligand superfamily member 12 | Y | Y |
| 664 | NM_012870 | tumor necrosis factor receptor superfamily, member 11b (osteoprotegerin) | Y | Y |
| 665 | AI169601 | tumor necrosis factor receptor superfamily, member 14 (herpesvirus entry mediator) | Y | N |
| 666 | NM_013091 | tumor necrosis factor receptor superfamily, member 1a | Y | Y |
| 667 | NM_013049 | tumor necrosis factor receptor superfamily, member 4 | Y | N |
| 668 | AF148323 | type 1 tumor necrosis factor receptor shedding aminopeptidase regulator | Y | Y |
| 669 | BG376248 | tyrosylprotein sulfotransferase 1 | Y | N |
| 670 | NM_053299 | ubiquitin D | Y | N |
| 671 | BI286141 | UDP-Gal:betaGlcNAc beta 1,4- galactosyltransferase, polypeptide 1 (mapped) | Y | Y |
| 672 | AA818125 | vacuolar protein sorting 33A (yeast) | Y | N |
| 673 | U81160 | vacuolar protein sorting 45 (yeast) | Y | N |
| 674 | AI010272 | Vascular endothelial zinc finger 1 (predicted) | Y | Y |
| 675 | NM_012759 | vav 1 oncogene | Y | N |
| 676 | NM_019302 | v-crk sarcoma virus CT10 oncogene homolog (avian) | Y | N |
| 677 | NM_017218 | v-erb-b2 erythroblastic leukemia viral oncogene homolog 3 (avian) | Y | Y |
| 678 | AI411103 | vesicle-associated membrane protein, associated protein a | Y | Y |
| 679 | NM_012555 | v-ets erythroblastosis virus E26 oncogene homolog 1 (avian) | Y | N |
| 680 | BM384958 | vitamin K epoxide reductase complex, subunit 1 | Y | N |
| 681 | NM_019156 | vitronectin | Y | Y |
| 682 | AF268467 | voltage-dependent anion channel 1 | Y | N |
| 683 | NM_031355 | voltage-dependent anion channel 3 | Y | N |
| 684 | BI298314 | von Willebrand factor | Y | N |
| 685 | BF283772 | v-rel reticuloendotheliosis viral oncogene homolog A (avian) | Y | Y |
| 686 | NM_022631 | wingless-type MMTV integration site 5A | Y | Y |
| 687 | BF417262 | X-box binding protein 1 | Y | N |
| 688 | AW526081 | X-ray repair complementing defective repair in Chinese hamster cells 4 | Y | N |
| 689 | AW535358 | YTH domain family 2 (predicted) | Y | N |
| 690 | NM_054002 | zinc finger and BTB domain containing 7a | Y | N |
| 691 | NM_031615 | zinc finger protein 148 | Y | N |
| 692 | AB025017 | zinc finger protein 36 | Y | Y |
| 693 | BG379973 | zinc finger, DHHC domain containing 13 | Y | Y |
| 694 | AA943537 | zyxin | Y | Y |

* “Y” indicates signal detected above background for gene probeset in 20% or more of the chips.

^†^ “Y” indicates significant differential gene expression within the Sham, SBR50, SBR50/IL-6, and SBR50/IL-6/G groups using False Discovery Rate (FDR) = 10%. “N” indicates not significant differential gene expression. “NA” indicates genes not included in the analysis because of not being detected in at least 20% of the chips.
