# Supplementary material for: IL-6-Mediated Activation of Stat3α Prevents Trauma/Hemorrhagic Shock-Induced Liver Inflammation
Source: PLoS One. 2011 Jun 29;6(6):e21449. doi: 10.1371/journal.pone.0021449 (PMC3127578; doi:10.1371/journal.pone.0021449)
Supplement: Table S2 — Inflammation transcriptome genes differentially expressed in the SBR50 vs. SHAM comparison. Table S2 identifies the 235 members of the inflammasome whose expression was altered among the experimental groups and describes the pattern of dysregulation between and among experimental groups in comparison to the SBR50 vs. Sham animals. Genes are grouped based on direction of dysregulation, allowing for identification of inflammasome genes dysregulated by our trauma/hemorrhagic shock model that are “normalized” by IL-6 and identification of inflammasome genes whose altered expression is, in part, regulated through Stat3. Group 1A represents genes increased in SBR50 vs. Sham and decreased in SBR50/IL-6 vs. SBR50. Group 1B represents genes increased in SBR50 vs. Sham and unchanged in SBR50/IL-6 vs. SBR50. Group 2A presents genes decreased in SBR50 vs. Sham and increased in SBR50/IL-6 vs. SBR50. Group 2B presents genes decreased in SBR50 vs. Sham and unchanged in SBR50/IL-6 vs. SBR50. Group 3 represents genes increased in SBR50 vs. Sham and increased in SBR50/IL-6 vs. SBR50. *Genes listed in regular type are anti-inflammatory, while genes listed in italics are pro-inflammatory. †FDR: False discovery rate. (DOC) [file pone.0021449.s002.doc]

**Table S2**. Inflammation transcriptome genes differentially expressed in the SBR50 vs. SHAM comparison.

| **Accesion Number** | **Gene Name^*^** | **Gene Symbol** | **SBR50 vs. Sham** | | **SBR50/IL-6 vs. SBR50** | | **SBR50/IL-6/G vs. SBR50/IL-6** | |
| --- | --- | --- | --- | --- | --- | --- | --- | --- |
|  |  |  | **Fold**  **P/Sham** | **FDR^†^** | **Fold**  **IL-6/P** | **FDR^†^** | **Fold**  **G/IL-6** | **FDR^†^** |
| **GROUP IA GENES INCREASED IN SBR50 vs. SHAM AND DECREASED IN SBR50/IL-6 vs. SBR50** | | |  |  |  |  |  |  |
| NM_012620 | serine (or cysteine) peptidase inhibitor, clade E, member 1 | Serpine1 | 29.09 | 0.00 | 0.25 | 0.00 | 1.73 | 0.14 |
| NM_012580 | heme oxygenase (decycling) 1 | Hmox1 | 19.97 | 0.00 | 0.13 | 0.02 | 1.20 | 0.83 |
| NM_053883 | dual specificity phosphatase 6 | Dusp6 | 16.82 | 0.00 | 0.53 | 0.06 | 1.32 | 0.40 |
| D64048 | phosphatidylinositol 3-kinase, regulatory subunit, polypeptide 1 | Pik3r1 | 6.17 | 0.00 | 0.21 | 0.01 | 0.64 | 0.42 |
| NM_022849 | deleted in malignant brain tumors 1 | Dmbt1 | 3.64 | 0.02 | 0.38 | 0.08 | 1.51 | 0.45 |
| AA943537 | Zyxin | Zyx | 2.78 | 0.00 | 0.61 | 0.04 | 1.73 | 0.02 |
| AI411788 | protein phosphatase 2 (formerly 2A), regulatory subunit A (PR 65), beta isoform | Ppp2rlb | 2.63 | 0.00 | 0.39 | 0.00 | 1.55 | 0.04 |
| NM_017039 | protein phosphatase 2 (formerly 2A), catalytic subunit, alpha isoform | Ppp2ca | 2.33 | 0.00 | 0.59 | 0.02 | 1.76 | 0.01 |
| BE113920 | signal transducer and activator of transcription 3 | Stat3 | 2.33 | 0.09 | 0.38 | 0.07 | 1.57 | 0.38 |
| BG379973 | zinc finger, DHHC domain containing 13 | Zdhhc13 | 2.06 | 0.01 | 0.56 | 0.03 | 1.08 | 0.75 |
| BM383696 | poly A binding protein, cytoplasmic 4 | Pabpc4 | 1.61 | 0.00 | 0.53 | 0.00 | 0.48 | 0.00 |
| NM_017040 | protein phosphatase 2 (formerly 2A), catalytic subunit, beta isoform | Ppp2cb | 1.60 | 0.00 | 0.64 | 0.00 | 0.98 | 0.87 |
| NM_021989 | tissue inhibitor of metalloproteinase 2 | Timp2 | 1.56 | 0.00 | 0.68 | 0.01 | 0.82 | 0.12 |
| *NM_053365* | *fatty acid binding protein 4, adipocyte* | *Fabp4* | *12.00* | *0.00* | *0.15* | *0.00* | *1.86* | *0.12* |
| *NM_012488* | *alpha-2-macroglobulin* | *A2m* | *10.11* | *0.01* | *0.15* | *0.03* | *2.11* | *0.38* |
| *NM_012591* | *interferon regulatory factor 1* | *Irf1* | *9.32* | *0.00* | *0.39* | *0.00* | *1.33* | *0.30* |
| *U13253* | *fatty acid binding protein 5, epidermal* | *Fabp5* | *9.32* | *0.00* | *0.32* | *0.01* | *1.08* | *0.83* |
| *NM_133306* | *oxidized low density lipoprotein (lectin-like) receptor 1* | *Olr1* | *9.02* | *0.00* | *0.30* | *0.00* | *2.01* | *0.03* |
| *AW531877* | *ephrin A1* | *Efna1* | *7.66* | *0.00* | *0.24* | *0.01* | *0.85* | *0.75* |
| *NM_053420* | *BCL2/adenovirus E1B 19 kDa-interacting protein 3* | *Bnip3* | *6.07* | *0.00* | *0.19* | *0.00* | *0.88* | *0.71* |
| *BI286141* | *UDP-Gal:betaGlcNAc beta 1,4- galactosyltransferase, polypeptide 1 (mapped)* | *B4galt1* | *5.12* | *0.00* | *0.29* | *0.00* | *1.05* | *0.87* |
| *NM_053612* | *heat shock 22kDa protein 8* | *Hspb8* | *5.08* | *0.00* | *0.36* | *0.01* | *1.30* | *0.43* |
| *U02315* | *neuregulin 1* | *Nrg1* | *5.08* | *0.00* | *0.43* | *0.06* | *0.70* | *0.41* |
| *AI411352* | *gap junction membrane channel protein alpha 1* | *Gja1* | *4.80* | *0.00* | *0.44* | *0.00* | *1.97* | *0.00* |
| *NM_013170* | *guanylate cyclase 2C* | *Gucy2c* | *3.98* | *0.00* | *0.29* | *0.00* | *3.04* | *0.00* |
| *NM_017192* | *endothelial differentiation, sphingolipid G-protein-coupled receptor, 5* | *Edg5* | *3.50* | *0.01* | *0.21* | *0.00* | *2.67* | *0.03* |
| *BI287978* | *growth arrest and DNA-damage-inducible 45 beta* | *Gadd45b* | *3.20* | *0.00* | *0.54* | *0.06* | *1.33* | *0.37* |
| *NM_019142* | *protein kinase, AMP-activated, alpha 1 catalytic subunit* | *Prkaa1* | *3.08* | *0.00* | *0.42* | *0.00* | *1.12* | *0.62* |
| *NM_021744* | *CD14 antigen* | *Cd14* | *3.07* | *0.00* | *0.49* | *0.04* | *0.73* | *0.35* |
| *BI284739* | *LPS-induced TN factor* | *Litaf* | *2.70* | *0.00* | *0.50* | *0.00* | *1.23* | *0.28* |
| *AB001382* | *secreted phosphoprotein 1* | *Spp1* | *2.67* | *0.01* | *0.45* | *0.05* | *2.08* | *0.06* |
| *NM_053843* | *Fc gamma receptor II beta* | *Fcer1g* | *2.64* | *0.00* | *0.52* | *0.01* | *1.71* | *0.03* |
| *BI283843* | *mitogen activated protein kinase kinase 3* | *Mapk3* | *2.61* | *0.00* | *0.65* | *0.06* | *0.90* | *0.64* |
| *NM_053843* | *Fc receptor, IgG, low affinity III /// Fc gamma receptor II beta* | *Fcgr3b* | *2.37* | *0.00* | *0.52* | *0.02* | *1.67* | *0.05* |
| *U72660* | *ninjurin 1* | *Ninj1* | *2.29* | *0.00* | *0.50* | *0.01* | *3.56* | *0.00* |
| *NM_053887* | *mitogen activated protein kinase kinase kinase 1* | *Mapk2k1* | *2.24* | *0.04* | *0.38* | *0.02* | *1.19* | *0.66* |
| *BE111083* | *mannan-binding lectin serine peptidase 1* | *Masp1* | *2.23* | *0.00* | *0.56* | *0.00* | *0.99* | *0.90* |
| *BI285141* | *CD24 antigen* | *Cd24* | *2.23* | *0.00* | *0.57* | *0.01* | *1.37* | *0.11* |
| *BF281278* | *inosine 5-monophosphate dehydrogenase 2* | *Impdh2* | *2.22* | *0.00* | *0.58* | *0.00* | *0.75* | *0.02* |
| *NM_031514* | *Janus kinase 2* | *Jak2* | *2.18* | *0.02* | *0.49* | *0.04* | *0.96* | *0.90* |
| *BI282724* | *arginine vasopressin-induced 1* | *Avpi1* | *2.17* | *0.00* | *0.65* | *0.03* | *2.02* | *0.00* |
| *NM_134432* | *angiotensinogen (serpin peptidase inhibitor, clade A, member 8)* | *Agt* | *2.11* | *0.00* | *0.43* | *0.00* | *1.31* | *0.22* |
| *NM_019905* | *annexin A2* | *Anxa2* | *2.10* | *0.00* | *0.72* | *0.09* | *1.95* | *0.00* |
| *AA875097* | *fibrinogen, alpha polypeptide* | *Fga* | *2.07* | *0.08* | *0.33* | *0.02* | *1.09* | *0.84* |
| *BM387008* | *caspase 3, apoptosis related cysteine protease* | *Casp3* | *2.00* | *0.00* | *0.54* | *0.00* | *1.46* | *0.03* |
| *AY066016* | *nuclear receptor subfamily 3, group C, member 1* | *Nr3c1* | *1.91* | *0.01* | *0.58* | *0.02* | *0.80* | *0.33* |
| *NM_012766* | *cyclin D3* | *Ccnd3* | *1.83* | *0.00* | *0.70* | *0.08* | *1.09* | *0.66* |
| *AF228684* | *adenosine A2a receptor* | *Adora2a* | *1.76* | *0.01* | *0.60* | *0.02* | *1.93* | *0.00* |
| *BG380414* | *chemokine (C-X-C motif) ligand 14* | *Cxcl14* | *1.74* | *0.01* | *0.55* | *0.01* | *2.23* | *0.00* |
| *NM_053619* | *complement component 5, receptor 1* | *C5ar1* | *1.73* | *0.00* | *0.51* | *0.00* | *1.84* | *0.00* |
| *NM_030833* | *interferon induced transmembrane protein 2 (1-8D)* | *Ifitm2* | *1.68* | *0.00* | *0.77* | *0.07* | *1.63* | *0.00* |
| *BF283772* | *v-rel reticuloendotheliosis viral oncogene homolog A (avian)* | *Rela* | *1.67* | *0.01* | *0.71* | *0.09* | *1.27* | *0.24* |
| *NM_013091* | *tumor necrosis factor receptor superfamily, member 1a* | *Tnfrsf1a* | *1.62* | *0.04* | *0.54* | *0.02* | *2.08* | *0.01* |
| *AI236590* | *myeloid differentiation primary response gene 88* | *Myd88* | *1.56* | *0.05* | *0.56* | *0.02* | *1.30* | *0.26* |
| *BM392321* | *homeodomain interacting protein kinase 2 (predicted)* | *Tp53* | *1.53* | *0.03* | *0.69* | *0.07* | *0.88* | *0.51* |
| *BF417479* | *24-dehydrocholesterol reductase* | *Dhcr24* | *1.51* | *0.02* | *0.69* | *0.04* | *0.63* | *0.01* |
| *AA893484* | *fibronectin 1* | *Flrt1* | *1.49* | *0.03* | *0.64* | *0.02* | *0.74* | *0.10* |
| *NM_053783* | *interferon gamma receptor 1* | *Ifngr1* | *1.48* | *0.03* | *0.55* | *0.01* | *1.18* | *0.35* |
| *NM_133307* | *protein kinase C, delta* | *Prkcd* | *1.42* | *0.03* | *0.73* | *0.06* | *1.23* | *0.20* |
| *NM_012696* | *kininogen 1 /// K-kininogen /// similar to alpha-1 major acute phase protein prepeptide* | *Kng1* | *1.42* | *0.02* | *0.68* | *0.02* | *1.84* | *0.00* |
| *AF209406* | *CD244 natural killer cell receptor 2B4* | *Cd244* | *1.40* | *0.01* | *0.66* | *0.01* | *1.12* | *0.39* |
| *BF407276* | *Rap guanine nucleotide exchange factor (GEF) 2 (predicted)* | *Rapgef2* | *1.38* | *0.05* | *0.66* | *0.02* | *0.84* | *0.28* |
| *NM_053866* | *phospholipase A2, activating protein* | *Plaa* | *1.32* | *0.05* | *0.71* | *0.03* | *1.10* | *0.51* |
| *NM_012559* | *fibrinogen, gamma polypeptide* | *Fgg* | *1.22* | *0.08* | *0.79* | *0.05* | *1.20* | *0.13* |
| *AI411103* | *vesicle-associated membrane protein, associated protein a* | *Vapa* | *1.21* | *0.06* | *0.81* | *0.05* | *1.33* | *0.01* |
| *NM_080888* | *BCL2/adenovirus E1B 19 kDa-interacting protein 3-like* | *Bnip3-like* | *1.19* | *0.09* | *0.73* | *0.01* | *0.60* | *0.00* |
|  |  |  |  |  |  |  |  |  |
| **GROUP 1B** | **GENES INCREASED IN SBR50 vs. SHAM AND UNCHANGED IN SBR50/IL-6 vs. SBR50** | |  |  |  |  |  |  |
| AI179507 | tissue factor pathway inhibitor 2 | Tfpi2 | 11.77 | 0.00 | 1.30 | 0.50 | 0.39 | 0.02 |
| NM_024385 | hematopoietically expressed homeobox | Hhex | 4.18 | 0.00 | 0.74 | 0.49 | 1.46 | 0.35 |
| NM_053819 | tissue inhibitor of metalloproteinase 1 | Timp1 | 2.66 | 0.00 | 0.82 | 0.52 | 1.96 | 0.02 |
| AI137406 | protein C receptor, endothelial | Procr | 2.36 | 0.01 | 1.20 | 0.63 | 0.91 | 0.76 |
| L12458 | Lysozyme | Lyz | 2.33 | 0.06 | 0.93 | 0.92 | 1.37 | 0.48 |
| AI227627 | CD9 antigen | Cd9 | 2.29 | 0.00 | 0.88 | 0.56 | 0.77 | 0.17 |
| BG671549 | superoxide dismutase 2, mitochondrial | Sod2 | 2.27 | 0.09 | 0.57 | 0.25 | 3.76 | 0.01 |
| U05989 | PRKC, apoptosis, WT1, regulator | Pawr | 2.06 | 0.00 | 1.35 | 0.17 | 0.99 | 0.97 |
| BI293600 | Solute carrier family 35, member B2 | Slc35b2 | 1.96 | 0.09 | 1.22 | 0.67 | 1.15 | 0.73 |
| BG666306 | Thrombomodulin | Thbd | 1.89 | 0.02 | 0.78 | 0.34 | 0.90 | 0.69 |
| NM_017200 | tissue factor pathway inhibitor | Tfpi | 1.50 | 0.05 | 0.80 | 0.28 | 0.74 | 0.15 |
| NM_012715 | Adrenomedullin | Adm | 1.48 | 0.06 | 1.12 | 0.64 | 1.76 | 0.02 |
| NM_017161 | adenosine A2B receptor | Adora2b | 1.48 | 0.03 | 0.79 | 0.19 | 2.57 | 0.00 |
| NM_133380 | interleukin 4 receptor, alpha | Il4ra | 1.35 | 0.00 | 0.96 | 0.68 | 1.28 | 0.01 |
| NM_022631 | wingless-type MMTV integration site 5A | Wnt5a | 1.07 | 0.09 | 0.99 | 0.93 | 0.96 | 0.28 |
| *NM_053587* | *S100 calcium binding protein A9 (calgranulin B)* | *S100a9* | *12.60* | *0.02* | *1.00* | *1.00* | *0.18* | *0.10* |
| *NM_053565* | *suppressor of cytokine signaling 3* | *Socs3* | *10.14* | *0.00* | *0.51* | *0.12* | *1.80* | *0.17* |
| *NM_031642* | *Kruppel-like factor 6* | *Klf6* | *9.50* | *0.00* | *0.78* | *0.67* | *0.87* | *0.77* |
| *BM384926* | *DnaJ (Hsp40) homolog, subfamily B, member 1 (predicted)* | *LOC681138* | *9.09* | *0.01* | *2.79* | *0.16* | *0.26* | *0.07* |
| *NM_053822* | *S100 calcium binding protein A8 (calgranulin A)* | *S100a8* | *7.70* | *0.01* | *1.08* | *0.95* | *0.14* | *0.02* |
| *NM_022194* | *interleukin 1 receptor antagonist* | *Il1rn* | *7.59* | *0.00* | *0.58* | *0.25* | *0.89* | *0.80* |
| *BM384099* | *N-myc downstream regulated gene 1* | *Ndrg1* | *5.90* | *0.00* | *0.54* | *0.17* | *1.58* | *0.30* |
| *NM_013060* | *inhibitor of DNA binding 2* | *Id2* | *5.69* | *0.00* | *0.69* | *0.20* | *1.77* | *0.05* |
| *NM_030845* | *chemokine (C-X-C motif) ligand 1* | *Cxcl1* | *5.28* | *0.01* | *0.87* | *0.87* | *0.60* | *0.41* |
| *NM_013144* | *insulin-like growth factor binding protein 1* | *Igfbp1* | *4.49* | *0.01* | *1.03* | *0.98* | *0.61* | *0.38* |
| *AF007789* | *plasminogen activator, urokinase receptor* | *Plaur* | *4.28* | *0.00* | *0.62* | *0.16* | *1.17* | *0.63* |
| *NM_012551* | *early growth response 1* | *Egr1* | *3.89* | *0.00* | *1.15* | *0.74* | *0.92* | *0.82* |
| *NM_053727* | *nuclear factor, interleukin 3 regulated* | *Nfil3* | *3.86* | *0.00* | *0.84* | *0.75* | *1.68* | *0.23* |
| *NM_053469* | *hepcidin antimicrobial peptide* | *Hamp* | *3.78* | *0.03* | *0.51* | *0.28* | *2.06* | *0.24* |
| *NM_030994* | *integrin alpha 1* | *Itga1* | *3.74* | *0.02* | *0.41* | *0.11* | *1.65* | *0.37* |
| *U24174* | *cyclin-dependent kinase inhibitor 1A* | *Cdkn1a* | *3.15* | *0.02* | *0.58* | *0.25* | *1.45* | *0.43* |
| *NM_022934* | *DnaJ (Hsp40) homolog, subfamily A, member 1* | *Jdp1* | *2.74* | *0.02* | *1.76* | *0.18* | *0.64* | *0.29* |
| *NM_012699* | *DnaJ (Hsp40) homolog, subfamily B, member 9* | *Dnajb9* | *2.53* | *0.02* | *0.55* | *0.14* | *0.55* | *0.14* |
| *AA957410* | *non-catalytic region of tyrosine kinase adaptor protein 2 (predicted)* | *Nck2* | *2.45* | *0.00* | *0.78* | *0.33* | *0.51* | *0.02* |
| *NM_057194* | *phospholipid scramblase 1* | *Plscr1* | *2.42* | *0.02* | *0.78* | *0.52* | *1.00* | *1.00* |
| *NM_012967* | *intercellular adhesion molecule 1* | *Icam1* | *2.26* | *0.03* | *0.95* | *0.92* | *1.00* | *1.00* |
| *U27767* | *regulator of G-protein signaling 4* | *Rgs4* | *2.15* | *0.02* | *0.77* | *0.46* | *2.80* | *0.01* |
| *AI411693* | *similar to immunoglobulin heavy chain 6 (Igh-6) /// similar to Ig H-chain V-region precursor /// similar to single chain Fv antibody fragment scFv 7-10A /// similar to Ig heavy chain V region MC101 precursor* | *RGD1359202* | *1.97* | *0.00* | *1.04* | *0.90* | *1.08* | *0.72* |
| *NM_012953* | *fos-like antigen 1* | *Fosl1* | *1.84* | *0.07* | *0.65* | *0.21* | *0.82* | *0.57* |
| *NM_012904* | *annexin A1* | *Anxa1* | *1.82* | *0.04* | *0.97* | *0.95* | *0.78* | *0.39* |
| *NM_053669* | *adaptor protein with pleckstrin homology and src homology 2 domains* | *Sh2b2* | *1.69* | *0.03* | *1.23* | *0.41* | *0.55* | *0.02* |
| *NM_013151* | *plasminogen activator, tissue* | *Plat* | *1.68* | *0.01* | *0.91* | *0.67* | *1.14* | *0.48* |
| *NM_012523* | *CD53 antigen* | *Cd53* | *1.66* | *0.01* | *0.73* | *0.12* | *1.14* | *0.50* |
| *NM_017260* | *arachidonate 5-lipoxygenase activating protein* | *Alox5ap* | *1.65* | *0.08* | *0.93* | *0.86* | *0.58* | *0.07* |
| *AA945737* | *chemokine (C-X-C motif) receptor 4* | *Cxcr4* | *1.49* | *0.06* | *0.88* | *0.58* | *1.62* | *0.03* |
| *NM_020542* | *chemokine (C-C motif) receptor 1* | *Ccr1* | *1.48* | *0.03* | *0.96* | *0.86* | *1.52* | *0.03* |
| *AI178808* | *interleukin 2 receptor, gamma (severe combined immunodeficiency)* | *Il2rg* | *1.40* | *0.06* | *1.13* | *0.54* | *2.11* | *0.00* |
| *NM_022214* | *chemokine (C-X-C motif) ligand 5* | *Cxcl5* | *1.40* | *0.07* | *0.92* | *0.72* | *0.69* | *0.05* |
| *BE116857* | *apoptotic chromatin condensation inducer 1* | *Acin1* | *1.30* | *0.10* | *0.92* | *0.64* | *1.14* | *0.40* |
| *AI411586* | *sequestosome 1* | *Sqstm1* | *1.29* | *0.05* | *0.94* | *0.72* | *1.21* | *0.14* |
| *NM_017104* | *colony stimulating factor 3 (granulocyte)* | *Csf3r* | *1.16* | *0.09* | *0.94* | *0.52* | *0.96* | *0.63* |
| *NM_012845* | *membrane-spanning 4-domains, subfamily A, member 2* | *Ms4a2* | *1.15* | *0.07* | *0.92* | *0.30* | *0.93* | *0.38* |
|  |  |  |  |  |  |  |  |  |
| **GROUP 2A** | **GENES DECREASED IN SBR50 vs. SHAM AND INCREASED IN SBR50/IL-6 vs. SBR50** | |  |  |  |  |  |  |
| U93307 | kinase insert domain protein receptor | Kdr | 0.19 | 0.00 | 3.33 | 0.00 | 0.73 | 0.34 |
| NM_054011 | SH3-domain binding protein 5 (BTK-associated) | Sh3bp5 | 0.36 | 0.00 | 1.68 | 0.06 | 1.10 | 0.73 |
| AA945624 | NAD(P)H dehydrogenase, quinone 2 | Nqo2 | 0.40 | 0.00 | 2.44 | 0.00 | 1.31 | 0.20 |
| BI296048 | myeloid-associated differentiation marker | Myadm | 0.42 | 0.00 | 2.03 | 0.01 | 1.83 | 0.01 |
| NM_012493 | alpha-fetoprotein | Afp | 0.50 | 0.01 | 2.02 | 0.01 | 0.86 | 0.52 |
| BG663097 | signal transducer and activator of transcription interacting protein 1 | Elp2 | 0.53 | 0.01 | 1.56 | 0.05 | 1.16 | 0.46 |
| AB038388 | Attractin | Atm | 0.56 | 0.01 | 1.96 | 0.01 | 0.47 | 0.00 |
| BI289536 | spastic paraplegia 21 homolog (human) | Spg21 | 0.57 | 0.00 | 1.55 | 0.01 | 1.50 | 0.01 |
| NM_031352 | drebrin-like | Dbnl | 0.63 | 0.05 | 1.61 | 0.06 | 2.64 | 0.00 |
| AF347936 | interleukin 1 receptor, alpha chain 1 | Il1ra1 | 0.65 | 0.01 | 1.60 | 0.01 | 1.27 | 0.14 |
| AW672589 | nuclear factor of kappa light chain gene enhancer in B-cells inhibitor, alpha | Nfkbia | 0.65 | 0.00 | 1.82 | 0.00 | 2.10 | 0.00 |
| U76206 | purinergic receptor P2Y, G-protein coupled, 14 | P2ry1 | 0.69 | 0.01 | 1.30 | 0.06 | 1.35 | 0.04 |
| NM_053481 | phosphatidylinositol 3-kinase, catalytic, beta polypeptide | Pik3cb | 0.72 | 0.03 | 1.34 | 0.06 | 0.90 | 0.48 |
| NM_024157 | complement factor I | Cfi | 0.76 | 0.00 | 1.21 | 0.01 | 0.57 | 0.00 |
| BG377057 | suppression of tumorigenicity 13 | St13 | 0.76 | 0.02 | 1.31 | 0.03 | 1.08 | 0.51 |
| NM_031640 | plasma glutamate carboxypeptidase | Pgcp | 0.79 | 0.03 | 1.31 | 0.02 | 0.35 | 0.00 |
| AI409930 | B-cell receptor-associated protein 31 | Bcap31 | 0.81 | 0.01 | 1.28 | 0.01 | 1.08 | 0.32 |
| NM_017050 | superoxide dismutase 1 | Sod1 | 0.91 | 0.01 | 1.15 | 0.00 | 0.94 | 0.09 |
| *BF283398* | *chemokine (C-X-C motif) ligand 12* | *Cxcl12* | *0.33* | *0.00* | *2.41* | *0.00* | *0.97* | *0.70* |
| *BM388891* | *interferon-induced protein 35* | *Ifi35* | *0.34* | *0.00* | *3.23* | *0.00* | *3.87* | *0.00* |
| *BG381669* | *tetratricopeptide repeat domain 7* | *Ttc7* | *0.42* | *0.00* | *1.77* | *0.02* | *1.23* | *0.35* |
| *BI284400* | *ajuba homolog (Xenopus laevis)* | *Jub* | *0.43* | *0.06* | *2.21* | *0.08* | *1.52* | *0.36* |
| *BI285347* | *complement component 4a /// complement component 4, gene 2* | *C4a* | *0.47* | *0.00* | *1.62* | *0.00* | *1.97* | *0.00* |
| *NM_017300* | *bile acid-Coenzyme A: amino acid N-acyltransferase* | *Baat* | *0.48* | *0.00* | *2.65* | *0.00* | *1.21* | *0.26* |
| *NM_012870* | *tumor necrosis factor receptor superfamily, member 11b (osteoprotegerin)* | *Tnfrsf11b* | *0.50* | *0.00* | *1.99* | *0.01* | *1.23* | *0.35* |
| *BI275972* | *G protein-coupled receptor 89 (predicted)* | *Gpr89* | *0.55* | *0.00* | *1.94* | *0.00* | *0.84* | *0.30* |
| *BF283018* | *similar to RIKEN cDNA 2810451A06* | *RGD1311098* | *0.58* | *0.01* | *1.87* | *0.00* | *1.05* | *0.80* |
| *BI282224* | *DnaJ (Hsp40) homolog, subfamily A, member 3* | *Dnaja3* | *0.59* | *0.00* | *1.48* | *0.00* | *1.10* | *0.24* |
| *BF416408* | *ATPase, H+ transporting, lysosomal V0 subunit a isoform 2 (predicted)* | *Atp6v0a2* | *0.59* | *0.00* | *1.40* | *0.01* | *1.37* | *0.01* |
| *NM_053886* | *lectin, mannose-binding, 1* | *Lman1* | *0.60* | *0.00* | *1.31* | *0.08* | *0.60* | *0.00* |
| *NM_012931* | *breast cancer anti-estrogen resistance 1* | *Bcar1* | *0.60* | *0.02* | *1.65* | *0.03* | *1.25* | *0.30* |
| *NM_057146* | *complement component 9* | *C9* | *0.64* | *0.00* | *1.29* | *0.04* | *1.36* | *0.02* |
| *D88250* | *complement component 1, s subcomponent /// similar to complement component 1, s subcomponent (predicted)* | *C1s* | *0.65* | *0.00* | *1.24* | *0.05* | *1.56* | *0.00* |
| *NM_019363* | *aldehyde oxidase 1* | *Aox1* | *0.65* | *0.00* | *1.37* | *0.01* | *0.32* | *0.00* |
| *NM_012599* | *mannose binding lectin 1, protein A* | *Mbl1* | *0.68* | *0.01* | *1.44* | *0.02* | *1.66* | *0.00* |
| *NM_031975* | *Parathymosin* | *Ptms* | *0.70* | *0.00* | *1.56* | *0.00* | *1.26* | *0.02* |
| *NM_030836* | *type 1 tumor necrosis factor receptor shedding aminopeptidase regulator* | *Erap1* | *0.70* | *0.00* | *1.27* | *0.02* | *2.04* | *0.00* |
| *NM_022277* | *caspase 8* | *Casp8* | *0.70* | *0.01* | *1.32* | *0.06* | *1.92* | *0.00* |
| *AI639117* | *complement factor B* | *Cfb* | *0.70* | *0.00* | *1.45* | *0.01* | *2.01* | *0.00* |
| *NM_012741* | *K-kininogen* | *Kng2* | *0.71* | *0.00* | *1.31* | *0.01* | *1.12* | *0.22* |
| *U39208* | *cytochrome P450 4F6* | *Cyp4f6* | *0.71* | *0.02* | *1.68* | *0.00* | *0.48* | *0.00* |
| *NM_017264* | *proteasome (prosome, macropain) 28 subunit, alpha* | *Psme1* | *0.72* | *0.00* | *1.29* | *0.02* | *2.40* | *0.00* |
| *NM_017257* | *proteasome (prosome, macropain) 28 subunit, beta* | *Psme2* | *0.72* | *0.06* | *1.45* | *0.05* | *3.41* | *0.00* |
| *NM_013087* | *CD 81 antigen* | *Cd81* | *0.72* | *0.00* | *1.17* | *0.08* | *1.05* | *0.56* |
| *AI227785* | *DnaJ (Hsp40) homolog, subfamily C, member 8* | *Dnajc8* | *0.73* | *0.02* | *1.37* | *0.02* | *1.60* | *0.00* |
| *NM_053690* | *DnaJ (Hsp40) homolog, subfamily C, member 14* | *Dnajc14* | *0.74* | *0.05* | *1.32* | *0.07* | *1.24* | *0.16* |
| *AI011448* | *Notch gene homolog 2 (Drosophila)* | *Notch2* | *0.74* | *0.10* | *1.59* | *0.02* | *1.18* | *0.37* |
| *NM_053785* | *transmembrane 4 superfamily member 4* | *Tm4sf4* | *0.75* | *0.08* | *1.78* | *0.00* | *1.81* | *0.00* |
| *NM_024131* | *D-dopachrome tautomerase* | *Ddt* | *0.75* | *0.00* | *1.35* | *0.00* | *0.97* | *0.67* |
| *NM_130756* | *acyl-CoA thioesterase 8* | *Acot8* | *0.76* | *0.00* | *1.53* | *0.00* | *0.80* | *0.02* |
| *BG381670* | *myeloid leukemia factor 2 (predicted)* | *Mlf2* | *0.77* | *0.01* | *1.41* | *0.00* | *1.53* | *0.00* |
| *NM_012826* | *alpha-2-glycoprotein 1, zinc* | *Azgp1* | *0.77* | *0.00* | *1.21* | *0.00* | *0.95* | *0.38* |
| *NM_022501* | *cysteine-rich protein 2* | *Stac2* | *0.78* | *0.03* | *1.49* | *0.00* | *2.36* | *0.00* |
| *Y18566* | *mannan-binding lectin serine peptidase 2* | *Masp2* | *0.78* | *0.00* | *1.29* | *0.01* | *1.09* | *0.27* |
| *AA819870* | *complement component 8, beta polypeptide (mapped)* | *C8bp* | *0.79* | *0.02* | *1.28* | *0.03* | *0.80* | *0.03* |
| *NM_022232* | *DnaJ (Hsp40) homolog, subfamily C, member 3* | *Dnajc3* | *0.80* | *0.10* | *1.42* | *0.02* | *0.95* | *0.69* |
| *NM_016994* | *complement component 3* | *C3* | *0.81* | *0.00* | *1.12* | *0.01* | *0.99* | *0.81* |
| *NM_024352* | *Macrophage stimulating 1 (hepatocyte growth factor-like)* | *Mst1* | *0.83* | *0.04* | *1.28* | *0.02* | *0.84* | *0.06* |
| *BI300565* | *a disintegrin and metalloprotease domain 10* | *Adam10* | *0.88* | *0.01* | *1.09* | *0.08* | *0.99* | *0.81* |
|  |  |  |  |  |  |  |  |  |
| **GROUP 2B** | **GENES DECREASED IN SBR50 vs. SHAM AND UNCHANGED IN SBR50/IL-6 vs. SBR50** | |  |  |  |  |  |  |
| NM_022180 | hepatocyte nuclear factor 4, alpha | Hnf4a | 0.33 | 0.00 | 1.35 | 0.12 | 1.45 | 0.05 |
| BG666368 | signal transducer and activator of transcription 2 | Stat2 | 0.41 | 0.01 | 1.66 | 0.15 | 2.22 | 0.03 |
| NM_021676 | SH3/ankyrin domain gene 3 /// hypothetical gene supported by NM_021676 | Shank3 | 0.44 | 0.01 | 1.54 | 0.18 | 1.10 | 0.77 |
| AI231686 | Transcription elongation factor B (SIII), polypeptide 3 | Tceb3 | 0.45 | 0.00 | 0.97 | 0.91 | 1.64 | 0.03 |
| NM_032612 | signal transducer and activator of transcription 1 | Stat1 | 0.45 | 0.08 | 2.20 | 0.10 | 6.09 | 0.00 |
| AI237657 | ADP-ribosylation factor-like 6 interacting protein 2 | Arl6ip2 | 0.47 | 0.00 | 1.08 | 0.78 | 0.82 | 0.33 |
| NM_022957 | serine (or cysteine) peptidase inhibitor, clade A, member 5 | Serpina5 | 0.56 | 0.05 | 1.05 | 0.91 | 0.77 | 0.38 |
| AA819349 | sideroflexin 1 | Sfxn1 | 0.66 | 0.01 | 0.99 | 0.98 | 1.16 | 0.32 |
| NM_024382 | serine (or cysteine) peptidase inhibitor, clade D, member 1 | Serpind1 | 0.70 | 0.00 | 1.20 | 0.10 | 0.79 | 0.03 |
| U06230 | protein S (alpha) | Pros1 | 0.70 | 0.02 | 1.14 | 0.42 | 0.70 | 0.03 |
| AF244895 | similar to DnaJ (Hsp40) homolog, subfamily B, member 12 | Dnajb1 | 0.71 | 0.05 | 1.14 | 0.47 | 0.76 | 0.12 |
| NM_012803 | protein C | Proc | 0.72 | 0.01 | 1.18 | 0.13 | 0.70 | 0.00 |
| AW252817 | transcription elongation factor A (SII), 3 | Tcea3 | 0.73 | 0.07 | 1.27 | 0.18 | 1.24 | 0.23 |
| NM_130406 | Fas-associated factor 1 | Faf1 | 0.74 | 0.07 | 1.16 | 0.41 | 1.51 | 0.02 |
| NM_053739 | beclin 1 (coiled-coil, myosin-like BCL2-interacting protein) | Bcen1 | 0.75 | 0.04 | 1.17 | 0.26 | 1.11 | 0.45 |
| AI010272 | Vascular endothelial zinc finger 1 (predicted) | Vezf1 | 0.76 | 0.08 | 1.21 | 0.25 | 1.36 | 0.06 |
| NM_031030 | cyclin G associated kinase | Gak | 0.78 | 0.00 | 1.06 | 0.52 | 1.78 | 0.00 |
| NM_019156 | Vitronectin | Vtn | 0.89 | 0.00 | 1.06 | 0.12 | 1.13 | 0.00 |
| NM_131907 | ATPase, Ca++-sequestering | Atp2c1 | 0.92 | 0.04 | 1.04 | 0.43 | 0.99 | 0.76 |
| *NM_012842* | *epidermal growth factor* | *Egfr* | *0.32* | *0.00* | *1.29* | *0.21* | *0.59* | *0.02* |
| *NM_017218* | *v-erb-b2 erythroblastic leukemia viral oncogene homolog 3 (avian)* | *Erbb3* | *0.47* | *0.00* | *0.96* | *0.89* | *1.08* | *0.70* |
| *NM_080767* | *proteosome (prosome, macropain) subunit, beta type 8* | *Psmb8* | *0.50* | *0.07* | *1.91* | *0.10* | *8.40* | *0.00* |
| *AI410467* | *intercellular adhesion molecule 2* | *Icam2* | *0.51* | *0.00* | *1.39* | *0.10* | *1.23* | *0.30* |
| *AI713204* | *Monoglyceride lipase* | *Mgll* | *0.52* | *0.00* | *1.27* | *0.18* | *0.86* | *0.38* |
| *AB019366* | *poly (ADP-ribose) glycohydrolase* | *Parg* | *0.52* | *0.00* | *1.12* | *0.65* | *1.49* | *0.06* |
| *AJ243973* | *RT1 class Ib, locus S3* | *Rt1s3* | *0.54* | *0.05* | *1.38* | *0.33* | *5.55* | *0.00* |
| *AI411541* | *Prostaglandin E receptor 3 (subtype EP3)* | *Ptger3* | *0.56* | *0.07* | *1.31* | *0.46* | *0.38* | *0.01* |
| *M22359* | *Murinoglobulin 1 homolog (mouse)* | *Mug1* | *0.60* | *0.08* | *1.02* | *0.98* | *0.23* | *0.00* |
| *NM_017322* | *mitogen-activated protein kinase 9* | *Mapk9* | *0.60* | *0.01* | *1.15* | *0.50* | *1.24* | *0.28* |
| *NM_017079* | *CD1d1 antigen* | *Cd1d1* | *0.60* | *0.03* | *1.46* | *0.12* | *1.28* | *0.30* |
| *NM_017097* | *cathepsin C* | *Ctsc* | *0.62* | *0.00* | *1.24* | *0.12* | *0.52* | *0.00* |
| *NM_031621* | *linker of T-cell receptor pathways* | *Sh2b3* | *0.62* | *0.01* | *0.84* | *0.26* | *1.86* | *0.00* |
| *NM_022704* | *mannose binding lectin 2 (protein C)* | *Mbl2* | *0.63* | *0.10* | *1.30* | *0.37* | *0.55* | *0.04* |
| *NM_012789* | *dipeptidylpeptidase 4* | *Dppp4* | *0.64* | *0.01* | *1.28* | *0.11* | *0.56* | *0.00* |
| *BF283688* | *tumor necrosis factor ligand superfamily member 12* | *Tnfsf12* | *0.65* | *0.08* | *1.46* | *0.13* | *1.19* | *0.48* |
| *NM_053758* | *phospholipase C, epsilon 1* | *Plce* | *0.66* | *0.05* | *1.29* | *0.24* | *1.09* | *0.69* |
| *U24150* | *tuberous sclerosis 2* | *Tsc2* | *0.66* | *0.06* | *1.29* | *0.26* | *1.18* | *0.46* |
| *NM_017038* | *protein phosphatase 1A, magnesium dependent, alpha isoform* | *Ppm1a* | *0.69* | *0.00* | *1.12* | *0.18* | *1.13* | *0.15* |
| *NM_053777* | *mitogen activated protein kinase 8 interacting protein* | *Mapk8i* | *0.71* | *0.04* | *1.29* | *0.12* | *0.86* | *0.37* |
| *AI705656* | *lymphotoxin B receptor* | *Ltbr* | *0.72* | *0.05* | *0.91* | *0.61* | *1.94* | *0.00* |
| *AI176519* | *immediate early response 3* | *Ier3* | *0.73* | *0.06* | *1.32* | *0.11* | *1.16* | *0.38* |
| *BG673589* | *paxillin* | *Pxn* | *0.75* | *0.03* | *1.16* | *0.27* | *1.17* | *0.24* |
| *BG378032* | *CD2 antigen (cytoplasmic tail) binding protein 2 (predicted)* | *Cd2bp2* | *0.75* | *0.09* | *0.97* | *0.88* | *1.34* | *0.09* |
| *BM389261* | *interferon gamma inducible protein 30* | *Ifi30* | *0.76* | *0.01* | *1.10* | *0.38* | *1.07* | *0.51* |
| *BE113312* | *adaptor-related protein complex 3, beta 1 subunit (predicted)* | *Ap3b1* | *0.77* | *0.09* | *1.23* | *0.18* | *1.30* | *0.10* |
| *NM_012516* | *complement component 4 binding protein, alpha* | *C4bpa* | *0.78* | *0.00* | *1.08* | *0.38* | *1.70* | *0.00* |
| *X73371* | *Fc receptor, IgG, low affinity IIb* | *Fcgr2b* | *0.83* | *0.09* | *1.20* | *0.12* | *1.21* | *0.10* |
| *BI282847* | *Tryptophan hydroxylase 1* | *Tph1* | *0.86* | *0.09* | *1.13* | *0.20* | *0.97* | *0.74* |
| *AI180413* | *apolipoprotein H* | *Apoh* | *0.86* | *0.03* | *1.07* | *0.30* | *0.84* | *0.01* |
| *NM_023103* | *alpha-1-inhibitor III /// Murinoglobulin 1 homolog (mouse)* | *Mug1* | *0.94* | *0.09* | *0.98* | *0.72* | *0.68* | *0.00* |
|  |  |  |  |  |  |  |  |  |
| **GROUP 3** | **GENES INCREASED IN SBR50 vs. SHAM AND INCREASED IN SBR50/IL-6 vs. SBR50** | |  |  |  |  |  |  |
| BE107450 | Neuronal regeneration related protein | Nrep | 1.96 | 0.05 | 2.40 | 0.02 | 0.10 | 0.00 |
| *AI408948* | *carbonic anhydrase 2* | *Ca2* | *1.52* | *0.09* | *1.82* | *0.03* | *0.33* | *0.00* |
| *AI412355* | *CD83 antigen (predicted)* | *Cd83* | *1.69* | *0.01* | *1.71* | *0.02* | *1.01* | *0.94* |
| *AB025017* | *zinc finger protein 36* | *Zfp3612* | *1.70* | *0.00* | *1.57* | *0.00* | *0.85* | *0.24* |
| *AI104324* | *DnaJ (Hsp40) homolog, subfamily A, member 4* | *Dnaja4* | *2.22* | *0.07* | *2.16* | *0.09* | *0.54* | *0.16* |
| *AI169104* | *chemokine (C-X-C motif) ligand 4* | *Cxcl4* | *4.46* | *0.00* | *2.26* | *0.09* | *0.09* | *0.00* |
| *AI599423* | *growth arrest and DNA-damage-inducible 45 gamma* | *Gadd45g* | *8.66* | *0.00* | *2.50* | *0.00* | *0.80* | *0.39* |
| *NM_031971* | *heat shock 70kD protein 1A /// heat shock 70kD protein 1B (mapped)* | *Hspa1a* | *45.41* | *0.00* | *7.32* | *0.06* | *0.08* | *0.02* |

^*^Genes listed in regular type are anti-inflammatory, while genes listed in italics are pro-inflammatory.

^†^FDR: False discovery rate.
